# Mitophagy-dependent mitochondrial DNA release links ionizing radiation to immunogenic cell death in pancreatic cancer

**DOI:** 10.1038/s41420-026-03269-7

**Published:** 2026-07-30

**Authors:** Chenghao Li, Yanxian Ren, Yong Chen, Huaying Yu, Jintao Cui, Fei Wang, Jin Li, Ao Sun, Heng Zhou

**Affiliations:** 1 School of Traditional Chinese Medicine, Faculty of Medicine, Yangzhou University, Yangzhou, 225009 China; 2 Department of General Surgery, The First Hospital of Lanzhou University, Lanzhou University, Lanzhou, 730000 China; 3 Department of Radio-Chemotherapy, Affiliated Hospital of Yangzhou University, Yangzhou University, Yangzhou, 225002 China; 4 School of Basic Medical Sciences & School of Public Health, Faculty of Medicine, Yangzhou University, Yangzhou, 225009 China

**Keywords:** Mitophagy, Immune cell death

## Abstract

Ionizing radiation (IR) elicits both cytotoxic and immunomodulatory effects, yet the precise link between mitochondrial remodeling and immunogenic cell death (ICD) remains elusive. Here, we report that IR triggers robust mitophagy in pancreatic cancer cells, marked by mitochondrial damage, Parkin/BNIP3 upregulation, and increased autophagic flux. In parallel, IR induces key hallmarks of ICD, including calreticulin exposure, HMGB1 translocation, and the release of ATP and IFN-β. Notably, genetic or pharmacological inhibition of mitophagy attenuated this immunogenic profile, whereas mitophagy induction amplified it. We further demonstrate that mitophagy triggers the cytosolic release of mitochondrial DNA (mtDNA) to activate immune signaling, which can be reversed by blocking mtDNA replication. In murine models, Parkin overexpression enhanced mitophagy and ICD marker expression, leading to reduced tumor burden and prolonged survival following irradiation. Collectively, our findings indicate that mtDNA leakage downstream of mitophagy links mitochondrial remodeling to radiation-induced ICD, suggesting that modulation of mitochondrial dynamics may represent a strategy to enhance both radiosensitivity and the immunogenic potential of radiotherapy in pancreatic cancer.

## Introduction

Radiotherapy remains a foundational treatment for solid tumors, primarily exerting its effects by inducing DNA damage [1] or promoting the accumulation of reactive oxygen species (ROS) [2]. While effective in directly eliminating cancer cells, its clinical efficacy is often constrained by therapeutic resistance and systemic relapse [3]. Beyond its direct cytotoxicity, recent studies have demonstrated that ionizing radiation (IR) can trigger immunogenic cell death (ICD), a specialized modality of cell death that enhances immune recognition of dying tumor cells [4]. ICD is characterized by the spatio-temporal release of damage-associated molecular patterns (DAMPs), including ATP, high mobility group box 1 (HMGB1), and the surface exposure of calreticulin (CALR) [5]. These signals regulate dendritic cell activation and antigen presentation, thereby fostering immune-mediated tumor control [6]. This immunogenic potential provides a strategic opportunity to amplify the therapeutic efficacy of radiotherapy beyond localized tumor destruction [7].

Mitochondria are central regulators of cellular stress and death pathways in response to radiation [8]. Mitophagy, a selective autophagic process for the clearance of damaged mitochondria, is increasingly recognized as a pivotal component of the radiation response. Our previous work demonstrated that IR upregulates mitophagy-associated proteins Parkin and BNIP3, thereby enhancing mitophagic flux [8]. Pre-emptively elevating mitophagy prior to X-ray exposure exacerbates DNA damage, attenuates G2/M checkpoint arrest, and augments PARP1 cleavage, collectively driving increased tumor cell death [9]. In a murine melanoma tumor-bearing model, this approach significantly prolonged survival, suggesting a radiosensitizing role for mitophagy [10]. However, mitophagy plays a dual role in malignancy, acting as either a survival mechanism or a death-promoting process depending on the cellular context [11]. For example, in clear cell renal cell carcinoma and nasopharyngeal carcinoma, the STAT2/SLC27A3 axis facilitates resistance to tyrosine kinase inhibitors by inducing PINK1-dependent mitophagy [12, 13]. Conversely, in pancreatic cancer and melanoma models, radiation-induced mitophagy via the Parkin/BNIP3 axis augments tumor radiosensitivity [8, 10]. These observations underscore the context-specific and magnitude-dependent nature of the biological effects of mitophagy.

Despite this growing body of evidence, the mechanistic relationship between radiation-induced mitophagy and ICD remains poorly defined. Given that both processes are independently triggered by IR, a critical unresolved question is whether and how mitophagy modulates the immunogenic profile of dying tumor cells. Emerging evidence suggests that mitochondrial byproducts, particularly mitochondrial DNA (mtDNA), may act as endogenous DAMPs that amplify innate immune signaling associated with ICD [14]. Central to this response is the cGAS-STING (cyclic GMP-AMP synthase-stimulator of interferon genes) pathway, a potent cytosolic DNA-sensing machinery that orchestrates the production of Type I interferons (IFNs) [15, 16]. For instance, in breast cancer models, mitochondrial disruption has been shown to increase mtDNA release, subsequently activating cGAS-STING signaling and enhancing T-cell tumor infiltration [17]. These findings support the concept that mitochondria-derived signals and their downstream sensing cascades function as upstream regulators of tumor immunogenicity.

Mechanistically, mitophagy may contribute to ICD through several convergent routes: the elimination of damaged mitochondria destabilizes anti-apoptotic proteins [18]; lysosomal degradation generates ROS bursts that enhance endoplasmic reticulum stress [4, 19]; and specific mitochondrial lipids, such as cardiolipin, promote inflammasome activation [20]. Among these, mtDNA released into the cytosol during mitochondrial remodeling has emerged as a particularly potent immunostimulatory signal via the cGAS-STING axis [21]. In the present study, we investigate the mechanistic interplay between radiation-induced mitophagy and ICD. Specifically, we aim to determine whether mtDNA, released downstream of mitophagy, acts as a critical mediator of ICD following radiotherapy. While durable adaptive immune responses represent an essential consequence of ICD, this study focuses on the mitochondrial-to-innate signaling events that confer immunogenic properties to radiation-induced cell death. By uncovering this regulatory axis, our work seeks to provide a conceptual framework for exploiting mitophagy to amplify the immunogenic potential of radiotherapy and guide the development of novel, mitochondria-targeted radiosensitizers.

## Results

### Ionizing radiation induces G2/M arrest and suppresses pancreatic cancer cell proliferation

**Fig. 1:**
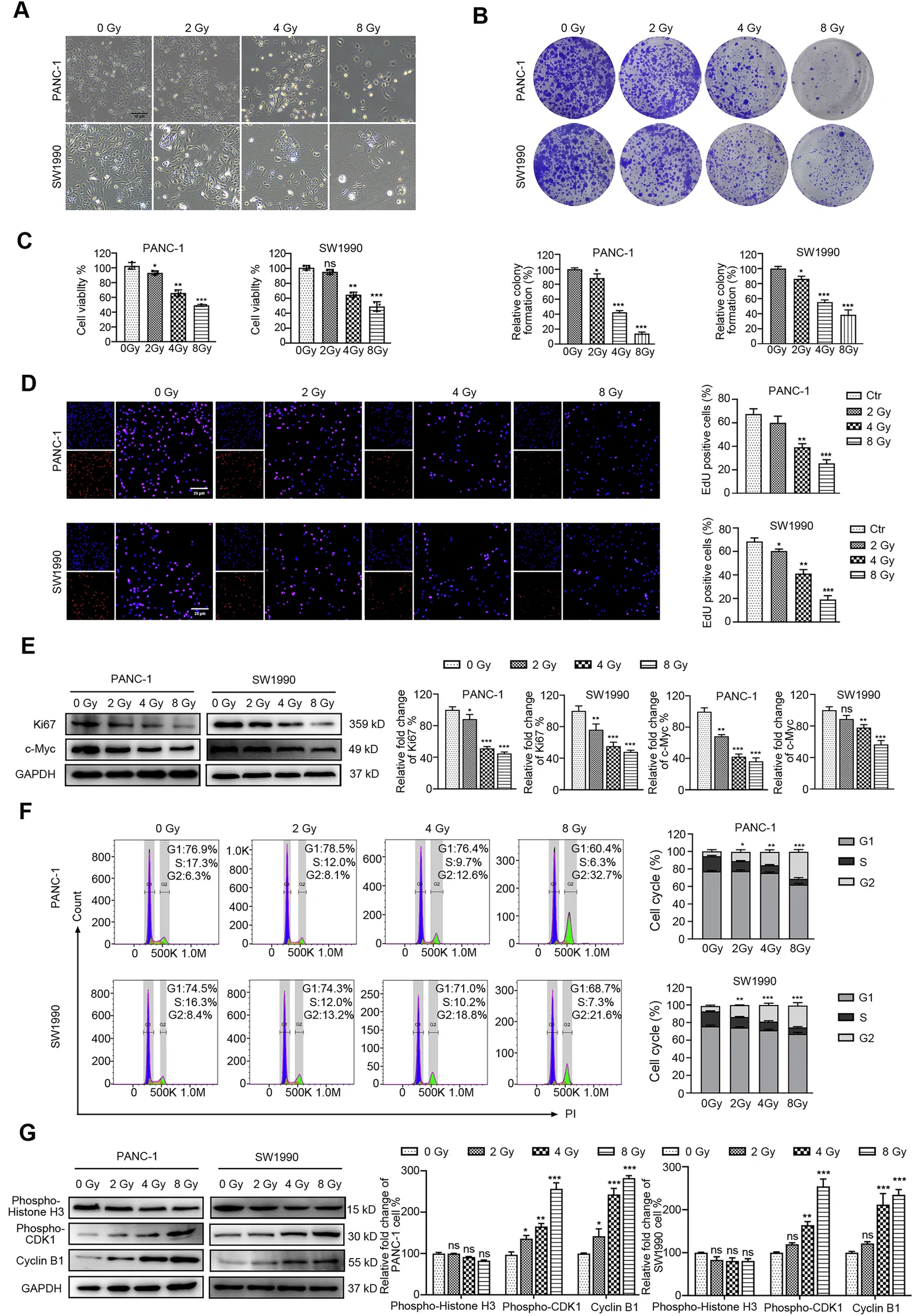
Ionizing radiation induces G2/M arrest and suppresses pancreatic cancer cell proliferation. **A** Morphological changes of PANC-1 and SW1990 cells 48 h after irradiation (0, 2, 4, 8 Gy). **B** Representative colony formation images and quantification. **C** Cell viability measured by CCK-8 assay. **D** Cell proliferation assessed by EdU incorporation. **E** Western blot analysis of proliferation markers Ki67 and c-Myc. **F** Flow cytometry analysis showing G2/M phase arrest after irradiation. **G** Western blot detection of cell cycle regulators p-Histone H3, p-CDK1, and Cyclin B1. Data are expressed as mean ± SD. *P < 0.05, **P < 0.01, ***P < 0.001.

To assess the impact of IR on pancreatic cancer growth, PANC-1 and SW1990 cells were exposed to increasing doses of X-ray irradiation (2, 4, and 8 Gy). Morphological examination at 48 h revealed that irradiated cells exhibited characteristic signs of stress, including cellular swelling and deformation (Fig. 1A). Proliferative capacity was markedly impaired in a dose-dependent manner, as evidenced by reduced colony formation (Fig. 1B), decreased metabolic activity in CCK-8 assays (Fig. 1C), and a decline in DNA synthesis shown by EdU incorporation (Fig. 1D). These findings were corroborated by Western blot analysis, which showed a substantial drop in the levels of proliferation markers Ki67 and c-Myc (Fig. 1E). Further analysis of cell cycle distribution via flow cytometry showed that IR caused cells to accumulate in the G2/M phase (Fig. 1F). Mechanistically, this arrest was characterized by increased inhibitory phosphorylation of CDK1 and accumulation of Cyclin B1, while levels of the mitotic marker phospho-histone H3 remained stable (Fig. 1G). These results indicate that IR activates the G2/M checkpoint, preventing cells from entering mitosis. Collectively, these data establish that IR induces a potent proliferative blockade, providing a baseline for investigating subsequent stress-induced cell death mechanisms.

### Ionizing radiation triggers mitophagy in pancreatic cancer cells

**Fig. 2:**
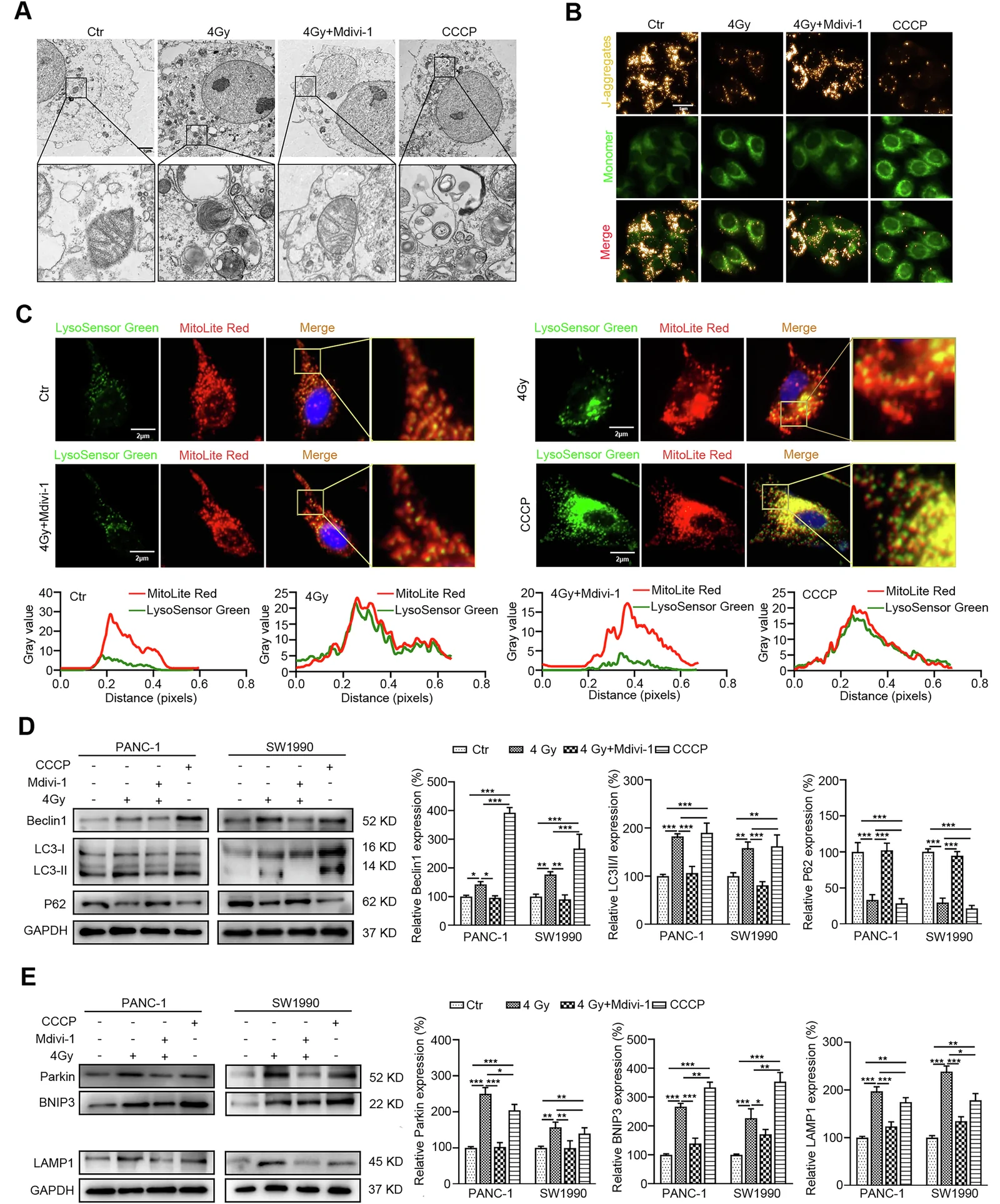
Ionizing radiation triggers mitophagy in pancreatic cancer cells. **A** Transmission electron microscopy images showing mitochondrial morphology after radiation, CCCP (1 μM), or Mdivi-1 (2 μM) treatment. **B** JC-10 assay showing mitochondrial membrane potential. **C** Co-localization of mitochondria (MitoTracker Red) and lysosomes (LysoSensor Green) indicating mitophagic activity. **D** Western blot analysis of Beclin-1, LC3, and p62. **E** Western blot detection of mitophagy regulators Parkin, BNIP3, and LAMP1. Data are expressed as mean ± SD. *P < 0.05, **P < 0.01, ***P < 0.001.

To determine whether IR-induced stress involves mitochondrial remodeling, we first examined the ultrastructure of PANC-1 cells using transmission electron microscopy. At 48 h post-irradiation (4 Gy), cells displayed clear signs of mitochondrial distress, including swelling, cristae disruption, and vacuolization, representing morphological features that mirrored those induced by the mitophagy activator CCCP. These structural alterations were largely prevented by the mitophagy inhibitor Mdivi-1 (Fig. 2A). In line with these morphological changes, JC-10 staining revealed significant mitochondrial membrane depolarization following IR or CCCP treatment, which was partially rescued by Mdivi-1 (Fig. 2B). To assess whether these damaged mitochondria were targeted for degradation, we performed mitochondrial–lysosomal co-localization analysis. IR exposure significantly enhanced the overlapping signals of MitoTracker Red and LysoSensor Green, indicating an accelerated mitophagic flux similar to that observed in CCCP-treated cells (Fig. 2C). This recruitment was substantially reduced upon Mdivi-1 treatment. Molecular analysis further confirmed autophagic activation, as IR and CCCP treatment increased Beclin-1 expression and the LC3-II/LC3-I ratio while promoting p62 degradation (Fig. 2D and Fig. S1A, B). Crucially, IR significantly upregulated the mitophagy-specific regulators Parkin and BNIP3, an effect that was blunted by Mdivi-1 (Fig. 2E). Overall, these findings demonstrate that IR triggers mitochondrial damage and activates the mitophagy pathway in pancreatic cancer cells.

### Ionizing radiation induces immunogenic cell death hallmarks in pancreatic cancer cells

**Fig. 3:**
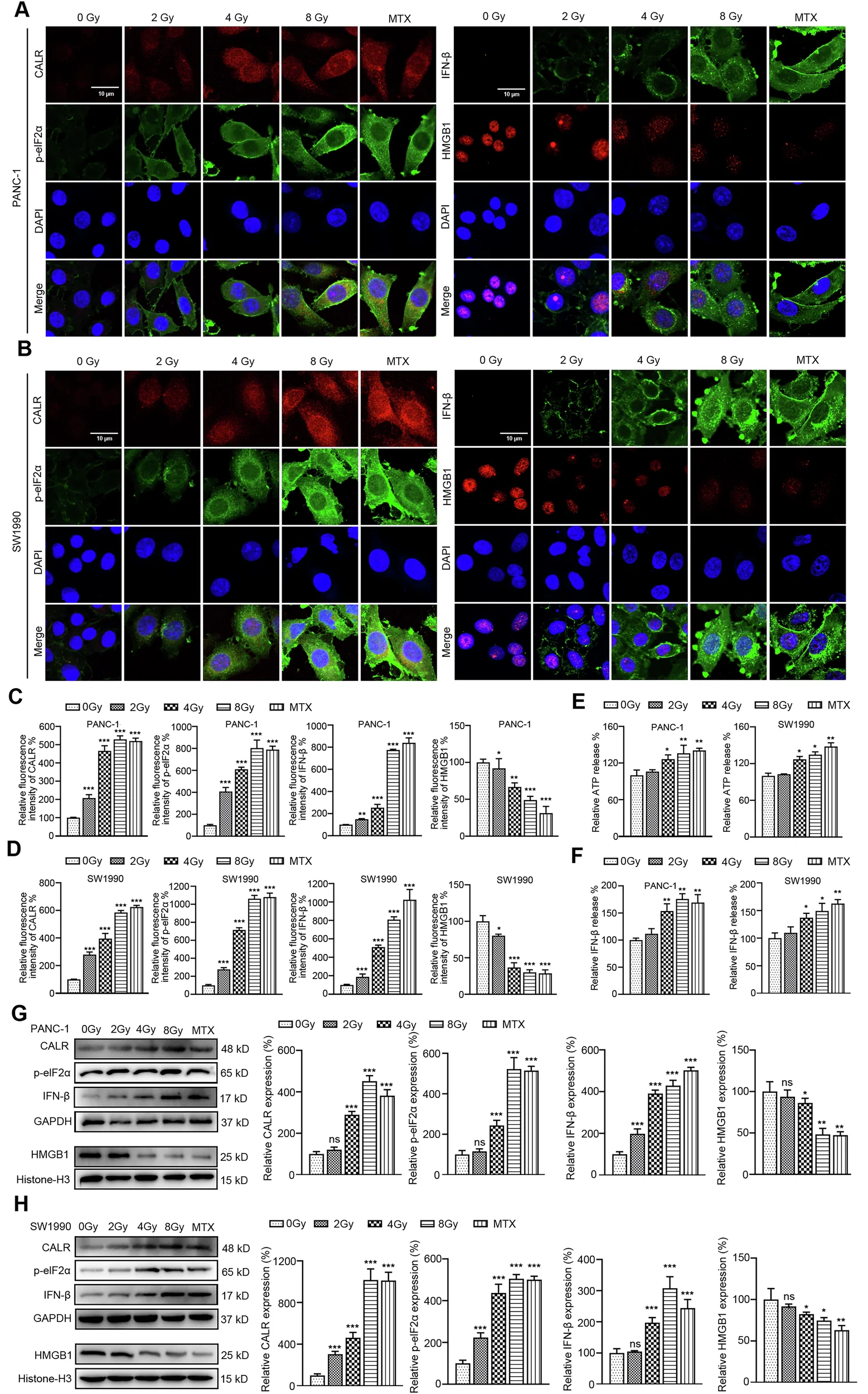
Ionizing radiation induces immunogenic cell death hallmarks in pancreatic cancer cells. **A**–**D** Immunofluorescence analysis showing CALR exposure, p-eIF2α expression, IFN-β expression, and HMGB1 translocation after irradiation or mitoxantrone (MTX, 1 µM) treatment. **E**, **F** ELISA quantification of ATP and IFN-β secretion. **G**, **H** Western blot analysis of CALR, p-eIF2α, IFN-β, and HMGB1. Data are expressed as mean ± SD. *P < 0.05, **P < 0.01, ***P < 0.001.

Given that IR inhibits cell growth and triggers mitophagy, we investigated whether radiation also evokes an ICD profile in pancreatic cancer cells. Immunofluorescence staining revealed that exposure to increasing doses of X-ray irradiation induced a series of ICD hallmarks comparable to those elicited by the canonical inducer mitoxantrone (MTX). Specifically, irradiated cells exhibited heightened surface exposure of CALR, increased phosphorylation of eIF2α, upregulated IFN-β, and prominent cytoplasmic translocation of HMGB1 (Fig. 3A–D). Consistent with these cellular changes, the extracellular concentrations of ATP and IFN-β in the culture supernatant increased in a dose-dependent manner (Fig. 3E, F). Molecular analysis in PANC-1 and SW1990 cells at 48 h post-irradiation further confirmed the upregulation of CALR, p-eIF2α, and IFN-β, alongside a marked reduction in nuclear HMGB1 abundance (Fig. 3G, H). Altogether, these findings indicate that IR triggers multiple signatures characteristic of ICD in pancreatic cancer cells.

### Mitophagy facilitates and amplifies radiation-induced immunogenic cell death

**Fig. 4:**
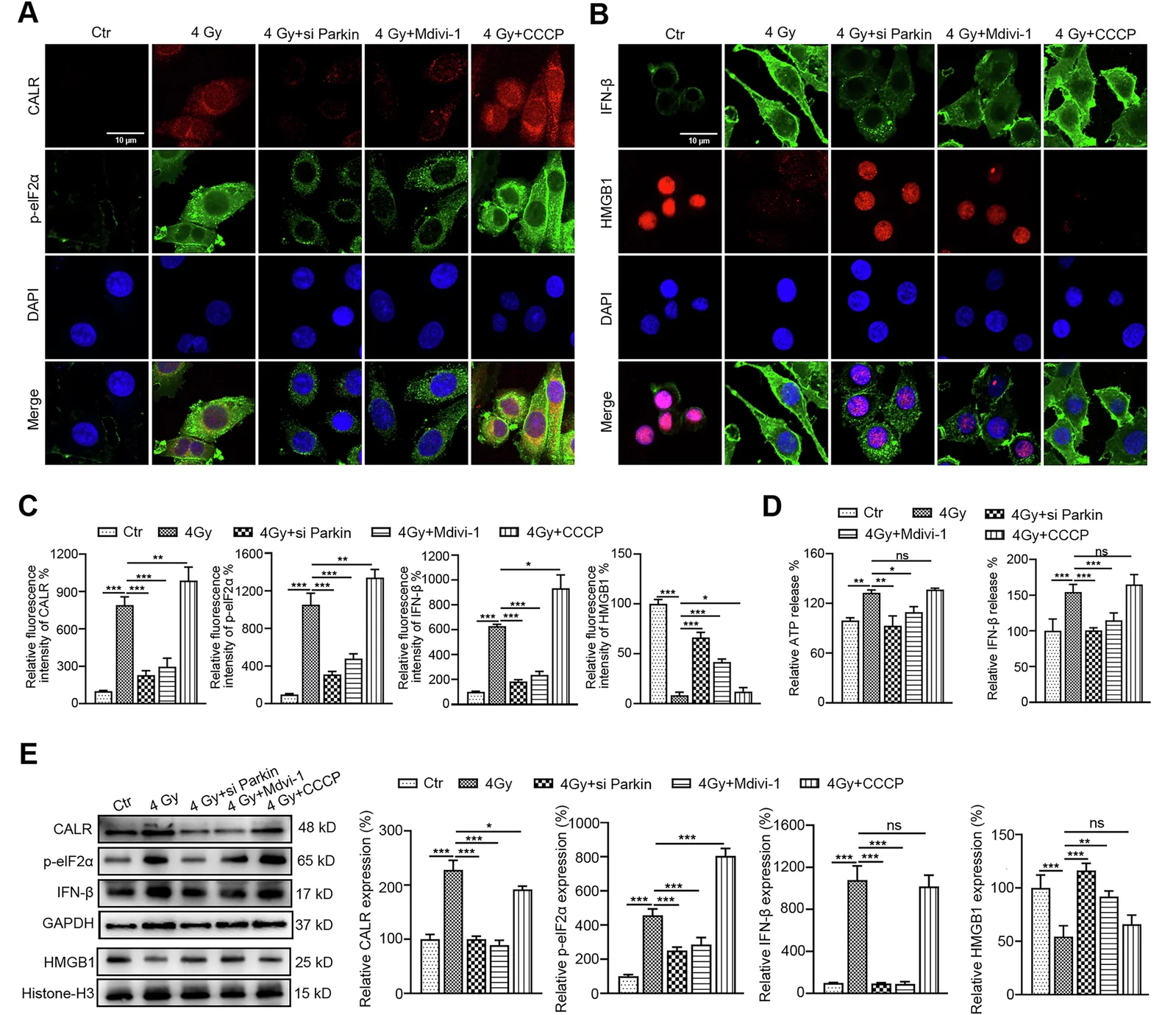
Mitophagy facilitates and amplifies radiation-induced immunogenic cell death. **A**–**C** Immunofluorescence staining showing CALR exposure, p-eIF2α levels, IFN-β expression, and HMGB1 localization in cells treated with radiation, Mdivi-1 (2 μM), CCCP (1 μM), or gene knockdown of Parkin. **D** ELISA quantification of ATP and IFN-β secretion following treatments. **E** Western blot validation of ICD-related markers under mitophagy modulation. Data are expressed as mean ± SD. *P < 0.05, **P < 0.01, ***P < 0.001.

The dual impact of IR on both cell fate and mitochondrial dynamics prompted us to investigate whether mitophagy drives the induction of ICD hallmarks. To test this, pancreatic cancer cells were exposed to 4 Gy irradiation following genetic or pharmacological modulation of mitophagy. Specifically, Parkin and BNIP3 were silenced using siRNAs (Fig. S1C, D), while Mdivi-1, CCCP were used to inhibit or induce mitophagic activity. Disruption of mitophagy, through either gene silencing or pharmacological inhibition, markedly attenuated the radiation-induced immunogenic profile. This was characterized by a significant reduction in surface calreticulin exposure, eIF2α phosphorylation, and IFN-β expression. Simultaneously, the radiation-induced translocation of HMGB1 from the nucleus to the cytoplasm was substantially blunted under these conditions. Conversely, co-treatment with CCCP amplified the induction of ICD hallmarks following irradiation (Fig. 4A–C and Fig. S2A–C). Consistent with these cellular changes, ELISA analysis demonstrated that mitophagy inhibition reduced the extracellular release of ATP and IFN-β, whereas mitophagy induction significantly enhanced their secretion (Fig. 4D and Fig. S2D). Western blot analysis further confirmed that genetic or pharmacological suppression of mitophagy reversed the radiation-induced expression patterns of key immunogenic proteins (Fig. 4E and Fig. S2E). Thus, these data demonstrate that mitophagy serves as a pivotal mediator that facilitates and amplifies radiation-induced ICD in pancreatic cancer cells.

### Mitophagy enhances radiation-induced immunogenic responses in vivo

**Fig. 5:**
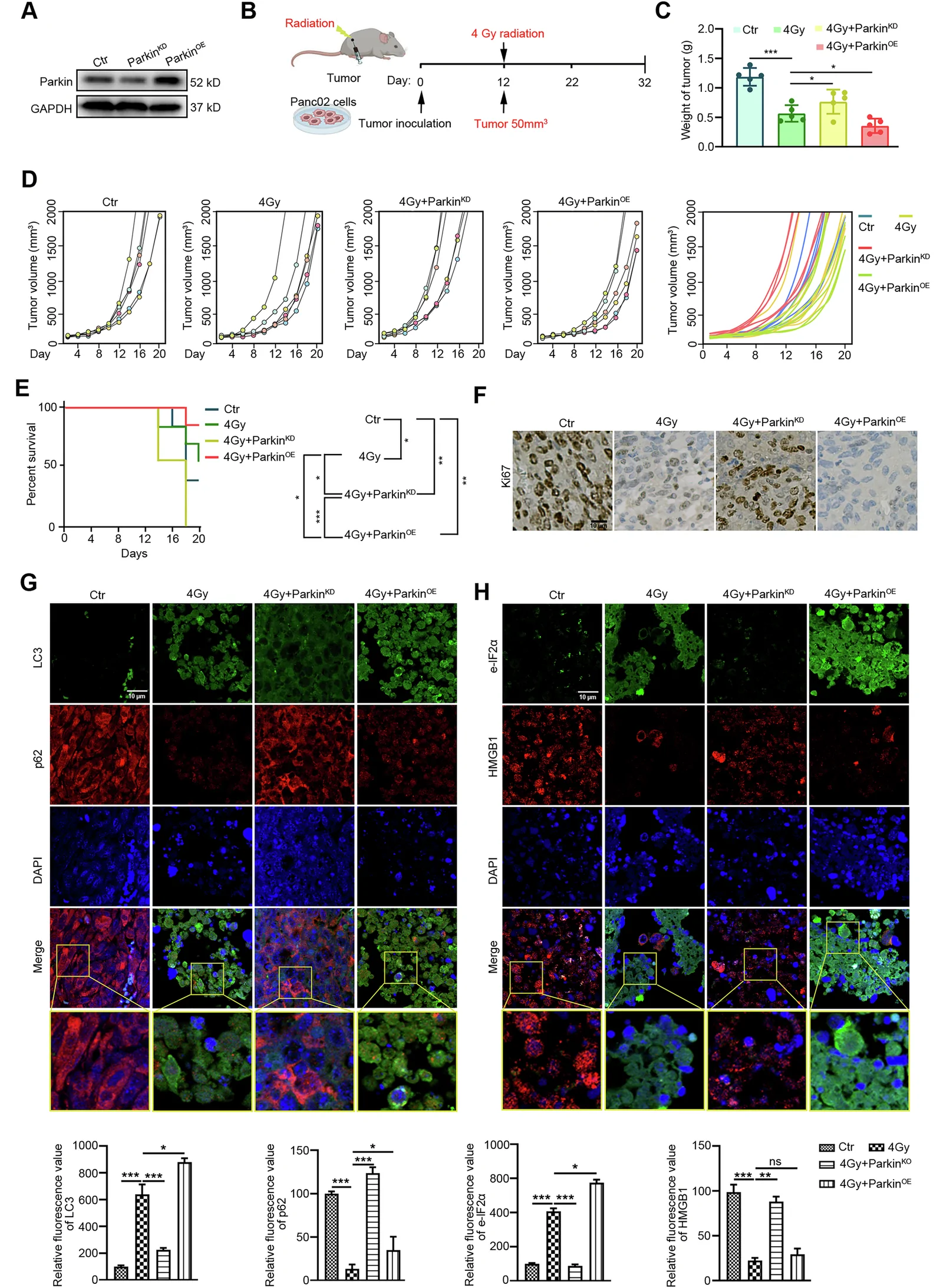
Mitophagy enhances radiation-induced immunogenic responses in vivo. **A** Schematic of Parkin knockdown/overexpression in Panc02 cells. **B** Experimental design of the murine subcutaneous tumor model. **C**–**E** Tumor weights, growth curves, and Kaplan–Meier survival analysis of irradiated mice bearing Parkin-modulated tumors. **F** Immunohistochemistry showing Ki67 expression. **G** Immunofluorescence analysis of LC3 and p62 expression. **H** Immunofluorescence detection of ICD markers p-eIF2α and HMGB1 translocation. *n* = 6, Data are expressed as mean ± SD. *P < 0.05, **P < 0.01, ***P < 0.001.

To validate the relationship between radiation-induced mitophagy and immunogenicity in vivo, we established a subcutaneous tumor model using murine Panc02 cells with Parkin knockdown or overexpression (Fig. 5A). These cells were implanted into immunocompetent C57BL/6 mice and treated with a single dose of 4 Gy X-ray irradiation once tumors reached approximately 50 mm³ (Fig. 5B). Following irradiation, mice bearing Parkin-overexpressing tumors exhibited significantly lower tumor weights (Fig. 5C), decelerated growth rates, smaller volumes (Fig. 5D), and extended survival (Fig. 5E) compared to control groups. Conversely, Parkin knockdown markedly blunted the therapeutic efficacy of radiation, leading to accelerated tumor progression. These differences were primarily observed following IR treatment, suggesting that Parkin-dependent mitophagy specifically modulates the radiotherapy response rather than intrinsic tumor growth. Immunohistochemical analysis of excised tissues showed a significant reduction in Ki67 expression in irradiated Parkin-overexpressing tumors, confirming an impaired proliferative capacity (Fig. 5F). Immunofluorescence staining further demonstrated increased LC3 levels and decreased p62 accumulation, confirming enhanced mitophagic activity in vivo (Fig. 5G). Crucially, tumors from the Parkin-overexpressing group displayed heightened eIF2α phosphorylation and prominent cytoplasmic translocation of HMGB1 post-irradiation, reflecting the robust induction of immunogenic signatures (Fig. 5H). We further found using bioinformatics analysis that pancreatic cancer patients with high expression of the autophagy-related gene Parkin (PRKN) exhibited longer survival, stronger immune infiltration in lesions, higher CD8^+^ T cell levels, and lower Treg cell levels (Fig. S3A–G). Consistent with these bioinformatics findings, irradiated Parkin-overexpressing mouse tumors showed a substantial increase in the infiltration of CD8^+^ T cells and elevated levels of IFN-γ, Granzyme B, and Perforin in both tumor tissues (Fig. S4A–D) and peripheral blood (Fig. S4E–H). These results indicate the activation of a robust systemic antitumor immune response. Taken together, these in vivo findings confirm that Parkin-dependent mitophagy augments radiation-induced antitumor efficacy by driving the expression of ICD hallmarks in pancreatic cancer.

### Mitophagy-dependent mtDNA leakage bridges radiation-induced stress and immunogenic cell death

**Fig. 6:**
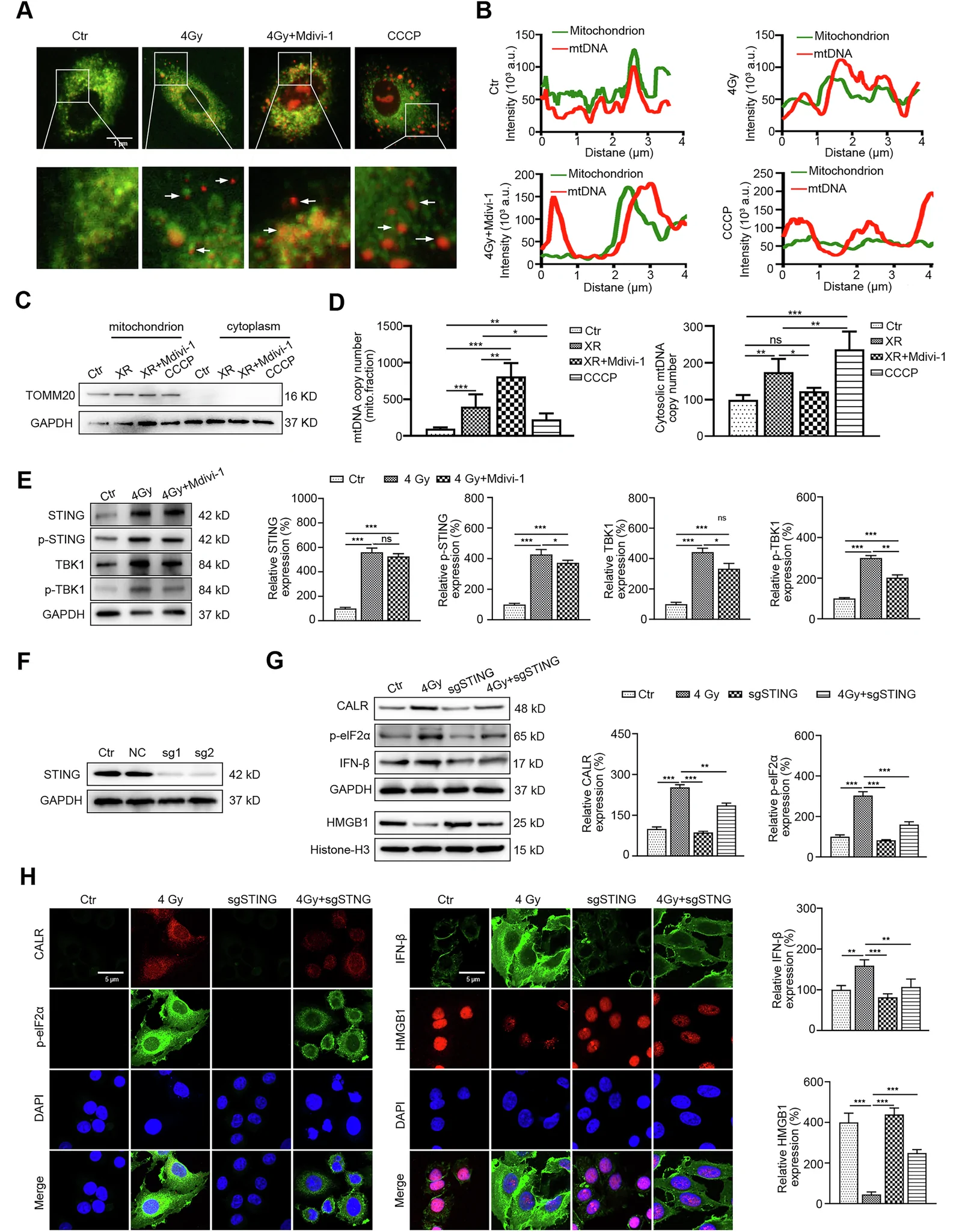
Ionizing radiation triggers mitophagic mtDNA release into the cytosol. **A** Representative immunofluorescence images of PANC-1 cells showing the subcellular localization of mtDNA (red) and mitochondria (MitoTracker, green) 48 h after treatment with 4 Gy irradiation, Mdivi-1, or CCCP. **B** Fluorescence co-localization analysis of mtDNA and mitochondria. **C** The isolation of mitochondrial and cytosolic fractions from PANC-1 cells. **D** Quantitative PCR analysis of mtDNA copy numbers in mitochondrial and cytosolic fractions following the indicated treatments. **E** Western blot analysis of total and phosphorylated STING and TBK1 levels in PANC-1 cells at 48 h post-treatment. **F** Western blot confirming reduced protein expression of STING following sgRNA transfection in PANC-1 cells. **G** Western blot analysis of ICD-associated markers including CALR, p-eIF2α, IFN-β, and HMGB1. **H** Representative immunofluorescence images showing the expression and localization of CALR, p-eIF2α, IFN-β, and HMGB1. Data are presented as mean ± SD from three independent experiments. *P < 0.05, **P < 0.01, ***P < 0.001.

**Fig. 7:**
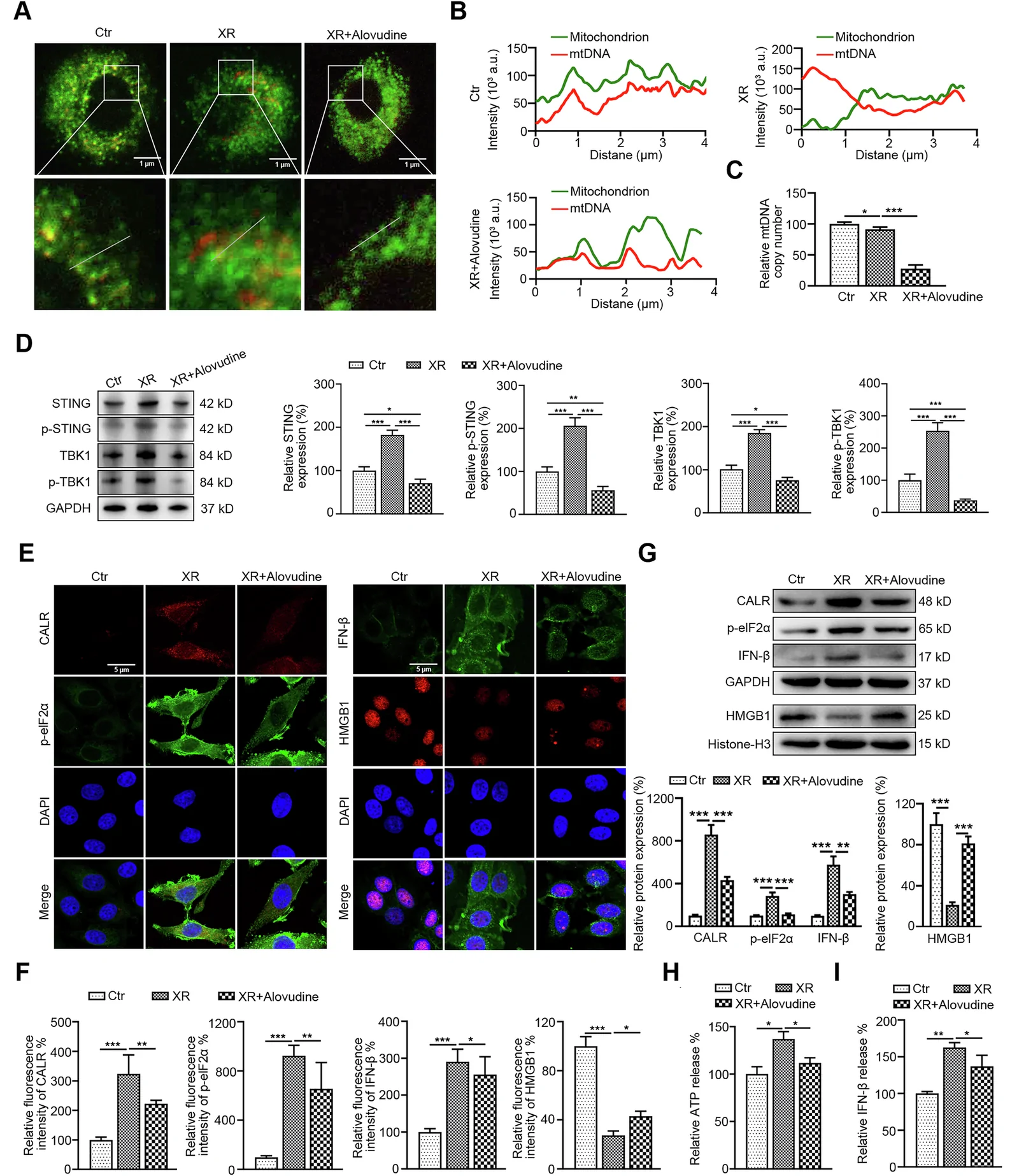
mtDNA leakage is essential for the induction of immunogenic cell death. **A** Representative fluorescence images of PANC-1 cells showing the effect of alovudine on radiation-induced cytosolic mtDNA accumulation. **B** Fluorescence co- localization analysis of mtDNA and mitochondria in PANC-1 cells following co-treatment with alovudine and irradiation. **C** Quantitative PCR analysis of cytosolic mtDNA levels in irradiated cells with or without alovudine treatment. **D** Western blot analysis showing the impact of alovudine on the radiation-induced phosphorylation of STING and TBK1. **E** Representative immunofluorescence images of surface CALR exposure, p-eIF2α expression, IFN-β expression, and HMGB1 cytoplasmic translocation in irradiated cells following alovudine treatment. **F**, **G** Western blot analysis of key immunogenic proteins, including CALR, p-eIF2α, IFN-β, and HMGB1 in PANC-1 cells under the indicated conditions. ELISA measurements of extracellular ATP (**H**) and IFN-β (**I**) concentrations in the culture supernatant. Data are expressed as mean ± SD. *P < 0.05, **P < 0.01, ***P < 0.001.

To delineate the mechanistic link between radiation-induced mitophagy and the resulting ICD profile, we investigated whether mtDNA released during mitochondrial remodeling serves as a critical mediator of this response. Immunofluorescence staining in PANC-1 cells revealed a partial redistribution of mtDNA from mitochondria to the cytosol 48 h after exposure to 4 Gy irradiation. Notably, CCCP treatment induced a more pronounced cytosolic accumulation of mtDNA, whereas Mdivi-1 treatment largely confined mtDNA within the mitochondria, effectively preventing cytosolic leakage (Fig. 6A, B). To quantify this distribution, mitochondrial and cytosolic fractions were isolated (Fig. 6C). Quantitative PCR analysis confirmed that mitochondrial mtDNA copy numbers remained significantly higher in the Mdivi-1 plus irradiation group compared to irradiation or CCCP treatment alone. Thus, cytosolic mtDNA levels surged following irradiation or CCCP exposure but were substantially reduced upon mitophagy inhibition (Fig. 6D). Consistent with the alterations in mtDNA localization, radiation treatment induced the upregulation and phosphorylation of the innate immune signaling proteins STING and TBK1. Importantly, mitophagy inhibition attenuated both total and phosphorylated levels of STING and TBK1, suggesting that mitophagic mtDNA release drives the activation of downstream innate immune pathways (Fig. 6E). In line with this, knockdown of STING (Fig. 6F) significantly attenuated the induction of cellular ICD by radiation (Fig. 6G, H). To verify the functional role of mtDNA, cells were treated with alovudine, an inhibitor of mtDNA replication. Alovudine significantly reduced cytosolic mtDNA accumulation, as confirmed by immunofluorescence and qPCR (Fig. 7A–C), and subsequently blunted the radiation-induced activation of STING and TBK1 (Fig. 7D). Furthermore, suppressing mtDNA release led to a significant attenuation of key immunogenic hallmarks, including reduced extracellular ATP and IFN-β levels, as demonstrated by western blot and ELISA (Fig. 7E–I). Therefore, we propose that mitophagy-dependent mtDNA leakage triggers innate immune signaling, which in turn orchestrates the induction of ICD signatures in pancreatic cancer cells.

## Discussion and conclusion

**Fig. 8:**
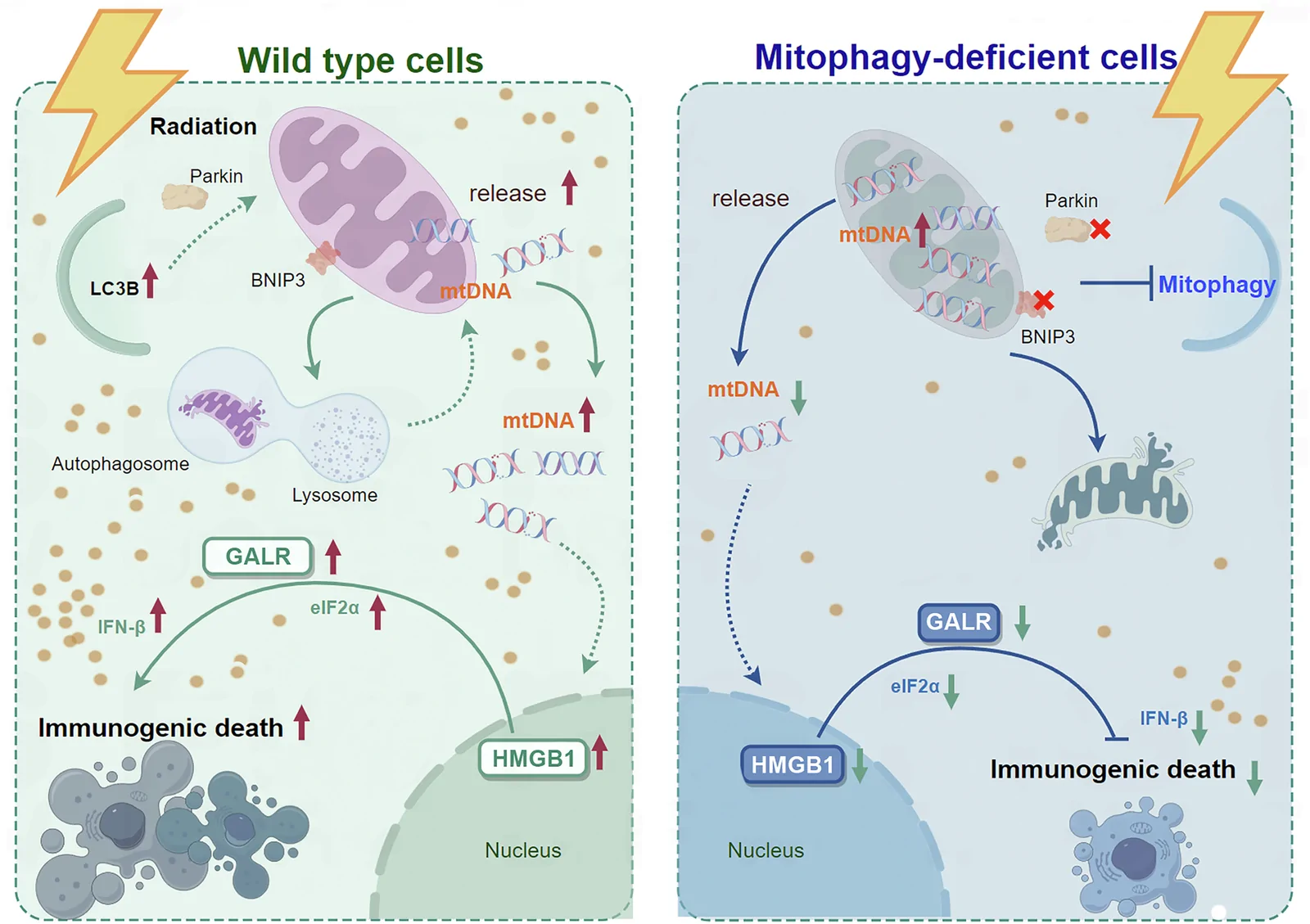
Schematic of the proposed mechanism. Ionizing radiation suppresses pancreatic cancer cell proliferation and induces mitophagy. Radiation-triggered mitophagy facilitates the release of mtDNA into the cytosol, where it acts as a danger-associated molecular pattern to promote ICD. This is characterized by calreticulin surface exposure, phosphorylation of eIF2α, HMGB1 nuclear export, and IFN-β/ATP release. Pharmacological or genetic inhibition of mitophagy prevents mtDNA leakage and attenuates ICD, whereas mitophagy induction enhances these responses. In vivo, tumors with elevated mitophagy display stronger ICD, reduced growth, and improved survival following irradiation. Collectively, these findings identify mitophagy-mediated mtDNA leakage as a key upstream event driving radiation-induced ICD and the subsequent antitumor immune response in pancreatic cancer.

Ionizing radiation has long been recognized as a potent tool for local tumor control. However, its broader therapeutic value increasingly resides in its capacity to trigger immunogenic signaling that supports systemic antitumor immunity [22, 23]. Central to this shift is the induction of ICD, which is a process where dying cancer cells release DAMPs to prime the immune system [24]. Our study extends this framework by identifying mitophagy as a significant regulator of radiation-induced immunogenicity. Specifically, we suggest that the leakage of mtDNA, a biological event occurring downstream of mitophagic activity, serves as a key immunostimulatory signal that links mitochondrial remodeling with the emergence of ICD hallmarks (Fig. 8).

Historically, mitophagy has been characterized primarily as a cytoprotective mechanism that preserves cellular homeostasis by clearing damaged mitochondria and curbing oxidative stress. For instance, PINK1-mediated mitophagy has been shown to alleviate myocarditis and aging-related inflammation by sequestering mtDNA and preventing cGAS–STING activation [25, 26]. Our findings, however, reveal a more complex role for this pathway in the context of radiotherapy. Contrary to the traditional view of mitophagy as a purely restrictive process, we show that radiation-induced mitophagic flux facilitates the cytosolic accumulation of mtDNA, which in turn amplifies ICD signaling. While pharmacological induction of mitophagy with CCCP potentiated the expression of ICD hallmarks such as surface CALR and p-eIF2α, inhibiting this process with Mdivi-1 effectively blunted these immunogenic features. These observations suggest that mitophagy does not simply eliminate mitochondrial contents but rather governs the spatial redistribution of potent immunostimulatory molecules.

This “mitophagy paradox”, in which a degradative pathway enhances the availability of a cytosolic ligand, potentially stems from the distinct kinetics of mitophagic flux under radiation stress [27]. Rather than proceeding to complete lysosomal digestion, radiation-induced mitophagy may function in a transitional or leaky manner. This allows mitochondrial contents to escape into the cytosol prior to terminal degradation. Our data support this model because while overall mitochondrial content decreases during mitophagic induction, the cytosolic pool of mtDNA surges. Conversely, preventing mitochondrial turnover through Mdivi-1 treatment sequesters mtDNA within the mitochondrial matrix, thereby silencing the immunogenic signal. These results highlight that the topological localization of mtDNA, rather than its absolute abundance, is likely the primary determinant of its immunogenic potency.

Furthermore, we establish that mtDNA is a functional mediator of ICD rather than a passive byproduct of cellular stress. By depleting the mtDNA pool with Alovudine, we were able to significantly reduce both its cytosolic accumulation and the subsequent induction of ICD hallmarks. This points to a potential requirement for radiation-induced immunogenicity, where the initiation of mitophagic machinery must be coupled with an available pool of mtDNA to fully engage innate immune pathways. Importantly, this mechanism translates to the in vivo setting. In our murine model, we not only confirmed the inhibitory effect of 4 Gy X-ray on pancreatic cancer but also clarified that Parkin-mediated mitophagy not only increased ICD markers but also remodeled the tumor microenvironment, leading to enhanced CD8^+^ T-cell infiltration and prolonged survival. These findings are consistent with our analysis of public databases, which showed that pancreatic cancer patients with high Parkin expression had better prognosis and stronger immune infiltration.

From a clinical standpoint, these findings provide a framework for improving treatment outcomes in immunologically “cold” tumors, especially pancreatic cancer. Specifically, targeted regulation of mitochondrial dynamics or the mitophagy-mtDNA axis offers a viable approach to sensitize these tumors to radiotherapy [28, 29]. The unique pathology of this malignancy has also spurred the development of various immunotherapies, including CAR-T cells and tumor vaccines combined with chemotherapy or checkpoint blockade [30, 31]. To optimize these interventions, integrated multi-omics analysis has become essential for identifying targets that improve chemotherapy response [32]. Looking ahead, exploring the synergy between radiotherapy and tumor vaccines in the context of mitochondrial stress may offer a transformative strategy for clinical management.

In summary, this work defines mitophagy not merely as a quality control process but as a context-dependent rheostat of tumor immunogenicity. By elucidating the mechanistic link between mitophagy and ICD, we provide a new framework that may assist in targeting mitochondrial turnover to maximize the immunogenic efficacy of radiotherapy.

## Materials and methods

### Chemicals and reagents

Mdivi-1 (PubChem CID: 3825829, purity ≥99.96%), CCCP (PubChem CID: 2603, purity ≥98.83%), Mitoxantrone (PubChem CID: 4212, purity ≥99.58%), and Alovudine (PubChem CID: 33039, purity ≥99.88%) were purchased from MedChemExpress (Princeton, NJ, USA). JC-10 and mitoLite Red were obtained from AAT Bioquest (Sunnyvale, CA, USA). LysoSensor Green, DAPI, and EdU Cell Proliferation Kit with Alexa Fluor 594 were purchased from MeiLun (Dalian, China). Mitochondria DNA-targeting tracker was obtained from Biolight Biotechnology (Guangzhou, China). ECL kit was purchased from ACE Biotechnology (Changzhou, China), and crystal violet staining solution from Beyotime (Shanghai, China). Dulbecco’s Modified Eagle’s Medium (DMEM) was obtained from Thermo Scientific (Waltham, MA, USA), and fetal bovine serum (FBS) from BioInd BI (Shanghai, China). Alexa Fluor 488 goat anti-mouse IgG (H + L) and Alexa Fluor 647 donkey anti-rabbit IgG (H + L) secondary antibodies were obtained from Abcam (Boston, MA, USA). Primary antibodies against STING, p-STING, TBK1, and p-TBK1 were obtained from ImmunoWay (CA, USA). Primary antibodies against Ki67, c-Myc, Phospho-Histone H3, Phospho-CDK1, Cyclin B1, Beclin 1, LC3B, P62, Parkin, BNIP3, LAMP1, CALR, HMGB1, IFN-β, Histone H3, and TOMM20 were purchased from HUABIO (Hangzhou, China).

### Cell culture

Human pancreatic ductal adenocarcinoma cell lines PANC-1 and SW1990 were obtained from Zhong Qiao Xin Zhou Biotechnology Co., Ltd. (Shanghai, China). Cells were cultured in DMEM supplemented with 10% FBS, 100 U/mL penicillin, and 100 μg/mL streptomycin, and maintained at 37 °C in a humidified incubator with 5% CO₂.

### Radiotherapy

X-rays were generated using an X-Rad 160 generator (Aolong, Dandong, China) with an energy of 100 kV and current of 13.0 mA. Cells or tumor-bearing mice anesthetized with pentobarbital sodium (50 mg/kg, i.p.) were placed in the irradiation chamber and exposed to X-rays. Customized lead plates were used to restrict irradiation to the tumor site and minimize exposure to surrounding tissues.

### Cell viability assay

Cell viability following irradiation was assessed using the Cell Counting Kit-8 (CCK-8; Dojindo, Japan). PANC-1 and SW1990 cells (5 × 10³ cells/well) were seeded into 96-well plates (Corning, MA, USA) and incubated overnight. Cells were then exposed to different doses of ionizing radiation (0, 2, 4, and 8 Gy) for 48 h. Subsequently, 10 μL of CCK-8 solution was added to each well and incubated for 30 min. Absorbance was measured at 450 nm using a microplate reader (Molecular Devices, CA, USA).

### Colony formation assay

Cells were treated with graded doses of ionizing radiation (0, 2, 4, and 8 Gy) for 48 h. They were then trypsinized, resuspended as single cells, and seeded into 35 mm culture dishes at a density of 300 viable cells per dish. After a 2-week incubation, colonies were fixed with methanol, stained with 0.5% crystal violet, washed, and photographed.

### Cell cycle assay

PANC-1 and SW1990 cells (1 × 10⁴ cells/well) were seeded in six-well plates, incubated overnight, and treated with ionizing radiation (0, 2, 4, or 8 Gy) for 48 h. Cells were then harvested and stained with propidium iodide (PI) using a commercial cell cycle staining kit (Multi, Hangzhou, China), following the manufacturer’s protocol. Cell cycle distribution was analyzed by flow cytometry (BD Biosciences, Franklin Lakes, NJ, USA).

### Transmission electron microscopy

Cells were fixed at 4 °C with 2.5% glutaraldehyde (electron microscopy grade), washed three times with PBS, and dehydrated in graded ethanol (50%, 70%, 90%). Samples were embedded in epoxy resin and polymerized at 45 °C and 60 °C. Ultrathin sections were cut, mounted on 200-mesh rhodium/copper grids, stained with uranyl acetate and lead citrate, and examined using a transmission electron microscope (Hitachi, Tokyo, Japan).

### Cell line construction

Parkin and BNIP3 knockdown, STING knockout, or Parkin overexpression was achieved using plasmids from MiaoLing Biotechnology (Wuhan, China). The specific sequences for these constructs are provided in Supplementary Table S1. Plasmids were transfected into cells with PolyFast transfection reagent (MCE, NJ, USA). After 72 h, stable clones were selected with puromycin disulfate (2 μg/mL; Topscience, Shanghai, China) and EGFP fluorescence. Western blot analysis was performed to confirm altered protein expression.

### Cell staining assay

Cells were seeded in 24-well plates (2 × 10³ cells/well) and treated with ionizing radiation or other specified conditions for 48 h. JC-10 dye was used to assess mitochondrial membrane potential. Mitochondrial autophagy was visualized using mitoLite Red and LysoSensor Green co-staining. Mitochondria and mtDNA were labeled with mitoLite Red and mitochondria DNA-targeting tracker, respectively. Images were acquired with a fluorescence microscope (Nikon, Japan), and co-localization was analyzed using ImageJ.

### ELISA assay

Levels of ATP and IFN-β in cell culture supernatants were measured using ELISA kits (Multi, Hangzhou, China), following the manufacturer’s protocols. Absorbance was read at 450 nm using a microplate reader (Molecular Devices, CA, USA).

### Mitochondria fractionation

Cells were cultured in 100 mm dishes until reaching ~1.5 × 10⁷ cells per condition. After washing with cold PBS, pellets were resuspended in isolation buffer (250 mM sucrose, 10 mM HEPES, 1 mM EDTA, pH 7.4), homogenized on ice with 200 strokes using a Dounce homogenizer, and centrifuged at 750 × *g* for 20 min. The supernatant was further centrifuged at 10,000 × *g* for 30 min to separate mitochondria (pellet) from cytoplasm (supernatant). Fraction purity was confirmed by immunoblotting.

### 4.12 qPCR assay

Mitochondrial and cytoplasmic DNA were extracted using a genomic DNA extraction kit (Accurate, Changsha, China). mtDNA levels were quantified using Power SYBR Green PCR Master Mix (Thermo Scientific) with specific primers (Invitrogen) on a StepOnePlus Real-Time PCR System (Thermo Scientific). Expression levels were normalized to ND1. Supplementary Table S2 lists the commercial primers and their corresponding sequence information.

### Western blot analysis

Cells were seeded in six-well plates (1 × 10⁴ cells/well) and exposed to ionizing radiation or other treatments. Proteins were extracted, separated by 12% SDS-PAGE, and transferred onto nitrocellulose membranes. Membranes were blocked with 5% non-fat milk in TBST for 2 h, incubated overnight at 4 °C with primary antibodies, and then with secondary antibody (goat anti-rabbit IgG, ImmunoWay, RS0002) for 1 h at room temperature. Bands were visualized with ECL reagents and imaged (Tanon, Shanghai, China). Densitometric analysis was performed using ImageJ.

### Immunofluorescence assay

Cells were seeded on sterile coverslips in 24-well plates (1 × 10⁴ cells/well). After treatment, cells were fixed with 4% paraformaldehyde, permeabilized with 0.5% Triton X-100, and incubated overnight at 4 °C with primary antibodies, followed by 2 h with fluorescent secondary antibodies. Nuclei were counterstained with DAPI. Imaging was performed using a fluorescence microscope (Olympus FV10-ASW2.1 Viewer, Japan).

### Experimental mouse model

Male C57BL/6 mice (8 weeks old) were obtained from the Animal Experiment Center of Yangzhou University and maintained under SPF conditions (12 h light/dark cycle, controlled temperature). All procedures were approved by the Animal Ethics Committee of Yangzhou University (Approval ID: YXYLL-2025-036; valid Dec 1, 2024–Dec 1, 2025) and complied with EU Directive 2010/63/EU. Mice were randomly divided into four groups (*n* = 6 per group). Panc02 cells (2 × 10⁶) were injected subcutaneously into the right axillary region. Two groups received untreated Panc02 cells, while the other two groups received Panc02 cells with Parkin overexpression or knockdown. After 12 days, when tumors reached ~100 mm³, they were irradiated with 4 Gy X-rays [8, 10]. Tumor growth and survival were monitored until ethical endpoints were reached. Mice were humanely euthanized in accordance with institutional guidelines.

### Flow cytometry assay

Peripheral blood was collected from mice via orbital venous plexus sampling, followed by the removal of erythrocytes using red blood cell lysis buffer. For immune profiling, the isolated leukocytes were first incubated with surface antibodies against CD3, CD4, and CD8. To detect intracellular cytokines and cytotoxic effector molecules, cells were fixed, permeabilized, and subsequently stained with antibodies against IFN-γ, Granzyme B, and Perforin according to the manufacturer’s protocols. Samples were processed using a BD FACSCelesta flow cytometer (USA). The frequencies of CD8⁺ T cell populations and their functional markers were analyzed using FlowJo software.

### Immunohistochemical assay

Tumor tissues were fixed in 10% neutral buffered formalin, embedded in paraffin, and cut into 4 μm sections. Following de-paraffinization and heat-induced antigen retrieval, the sections were incubated overnight at 4 °C with primary antibodies targeting CD8, IFN-γ, Granzyme B, and Perforin. Immune signals were detected using 3,3′-diaminobenzidine (DAB) substrate, and the slides were subsequently visualized and scanned using the TissueFAXS Viewer system (TissueGnostics, Vienna, Austria). For quantification, three representative fields were selected from each sample and analyzed using ImageJ software to determine the expression levels or cell counts of the target proteins.

### Bioinformatics analysis

All TMB (tumor mutational burden) values were normalized to the whole-exome size of 38 Mb in this study. The correlation between PRKN expression level and TMB score was examined. Patients were dichotomized into low- and high-PRKN expression subgroups based on the median PRKN expression value. Kaplan–Meier survival curves together with log-rank tests were used to compare survival differences between the two subgroups. The R packages *‘survival’* and *‘survminer’* were used for survival curve visualization. The relative proportions of 22 tumor-infiltrating immune cell subsets were quantified using the CIBERSORT algorithm. The composition of tumor-infiltrating immune cells was compared between the low- and high-PRKN expression groups. Pearson correlation analysis was performed to assess the associations between PRKN expression levels and the 22 immune cell subpopulations.

### Statistical analysis

All statistical analyses were performed using GraphPad Prism 9. Data are presented as mean ± standard deviation (SD) from at least three independent experiments. Comparisons were made using unpaired Student’s *t* test or one-way ANOVA with Dunnett’s post hoc test. Kaplan–Meier survival curves were analyzed using the log-rank test. A *p* value < 0.05 was considered statistically significant (*p < 0.05; **p < 0.01; ***p < 0.001).

## Supplementary information

Figure S1

Figure S2

Figure S3

Figure S4

Supplementary Figure Legends

Supplementary Table S1

Supplementary Table S2

Original western blot bands

## Data Availability

All data generated and analyzed during this study are included in this article. Each experiment was performed at least three times independently.

## References

[1] Poulton E, Desjardins A, Harding SM, Malbeteau L, Koritzinsky M. Cytosolic DNA in radiotherapy: relevance, mechanisms, and detection. Radiother Oncol. 2026;214:111220. 10.1016/j.radonc.2025.111220 41429720

[2] Forenzo C, Larsen J. Bridging clinical radiotherapy and space radiation therapeutics through reactive oxygen species (ROS)-triggered delivery. Free Radic Biol Med. 2024;219:88–103. 10.1016/j.freeradbiomed.2024.04.219 38631648

[3] Li H, Liu J, Jiao Y, Xu F, Wang S, Tang Q. Single-cell and spatial transcriptomics reveal P4HA2-mediated radiotherapy resistance mechanisms in breast cancer. Theranostics. 2026;16:2192–220. 10.7150/thno.121257 PMC12712805 41424847

[4] Yao J, Chen P, Xie D, Chen J, Lin X, Xu Y. Copper-induced cuproptosis and immunogenic cell death synergize with radiotherapy and PD-1 blockade in osteosarcoma. ACS Appl Mater Interfaces. 2025;17:46635–50. 10.1021/acsami.5c08888 40763959

[5] Galluzzi L, Guilbaud E, Schmidt D, Kroemer G, Marincola FM. Targeting immunogenic cell stress and death for cancer therapy. Nat Rev Drug Discov. 2024;23:445–60. 10.1038/s41573-024-00920-9 PMC11153000 38622310

[6] Galluzzi L, Kepp O, Zitvogel L, Tang D, Kroemer G. Cancer cell death: Cell-autonomous and immunogenic dimensions. Cancer Cell. 2026;44:281–305. 10.1016/j.ccell.2025.12.005 41478276

[7] He L, Ma J, Wu Y, Wang C, Lan T, Su L, et al. X-ray stimulates NQO1-dependent cascade reactions to induce strong immunogenicity for MRI-guided cancer radio-chemodynamic-immunotherapy. Theranostics. 2025;15:6768–88. 10.7150/thno.110573 PMC12203676 40585976

[8] Yang P, Li J, Zhang T, Ren Y, Zhang Q, Liu R, et al. Ionizing radiation-induced mitophagy promotes ferroptosis by increasing intracellular free fatty acids. Cell Death Differ. 2023;30:2432–45. 10.1038/s41418-023-01230-0 PMC10657348 37828085

[9] Deng H, Chen Y, Wang L, Zhang Y, Hang Q, Li P, et al. PI3K/mTOR inhibitors promote G6PD autophagic degradation and exacerbate oxidative stress damage to radiosensitize small cell lung cancer. Cell Death Dis. 2023;14:652. 10.1038/s41419-023-06171-7 PMC10558571 37802999

[10] Ren Y, Yang P, Li C, Wang WA, Zhang T, Li J, et al. Ionizing radiation triggers mitophagy to enhance DNA damage in cancer cells. Cell Death Discov. 2023;9:267. 10.1038/s41420-023-01573-0 PMC10382586 37507394

[11] Xia J, Jin J, Dai S, Fan H, Chen K, Li J, et al. Mitophagy: A key regulator of radiotherapy resistance in the tumor immune microenvironment. Mol Asp Med. 2025;105:101385. 10.1016/j.mam.2025.101385 40690876

[12] Lu D, Li Y, Niu X, Sun J, Zhan W, Shi Y, et al. STAT2/SLC27A3/PINK1-Mediated Mitophagy Remodeling Lipid Metabolism Contributes to Pazopanib Resistance in Clear Cell Renal Cell Carcinoma. Res (Wash D C). 2024;7:0539. 10.34133/research.0539 PMC11588985 39600540

[13] Zhang Y, Pang C, Zhang C, Wang Y, Wang P, Chen Y, et al. HILPDA-mediated lipidomic remodelling promotes radiotherapy resistance in nasopharyngeal carcinoma by accelerating mitophagy. Cell Mol Life Sci. 2023;80:242. 10.1007/s00018-023-04891-9 PMC11072511 37552373

[14] Yin M, Dong J, Sun C, Liu X, Liu Z, Liu L, et al. Raddeanin A enhances mitochondrial DNA-cGAS/STING axis-mediated antitumor immunity by targeting transactive responsive DNA-binding protein 43. Adv Sci (Weinh). 2023;10:e2206737. 10.1002/advs.202206737 PMC10161045 36876644

[15] Wang Q, Yu Y, Zhuang J, Liu R, Sun C. Demystifying the cGAS-STING pathway: precision regulation in the tumor immune microenvironment. Mol Cancer. 2025;24:178. 10.1186/s12943-025-02380-0 PMC12160120 40506729

[16] Wang H, Nie H, Zhao X, Chen K, Peng C, Zhang X, et al. Tumor-derived PRMT1 suppresses macrophage antitumor activity by inhibiting cGAS/STING signaling in gastric cancer cells. Cell Death Dis. 2025;16:649. 10.1038/s41419-025-07960-y PMC12381180 40858547

[17] Zhong N, Zu Z, Lu Y, Sha X, Li Y, Liu Y, et al. Mitochondria-targeted manganese-based mesoporous silica nanoplatforms trigger cGAS-STING activation and sensitize anti PD-L1 therapy in triple-negative breast cancer. Acta Biomater. 2025;199:374–86. 10.1016/j.actbio.2025.04.040 40294811

[18] Wu S, Ma X, Zhang X, Du K, Shi C, Almaamari AA, et al. Knockdown of NDUFAF6 inhibits breast cancer progression via promoting mitophagy and apoptosis. Cancer Biol Ther. 2025;26:2445220. 10.1080/15384047.2024.2445220 PMC11702941 39706687

[19] Vietri M, Miranda MR, Amodio G, Ciaglia T, Bertamino A, Campiglia P, et al. The link between endoplasmic reticulum stress and lysosomal dysfunction under oxidative stress in cancer cells. Biomolecules. 2025;15:930. 10.3390/biom15070930 PMC12292385 40723802

[20] Zhang S, Feng C, Yu N, Fang R, Zhang Y, Chen S, et al. Synthetic anticoagulant octaparin targets mitochondrial cardiolipin-GSDMD axis to rescue redox homeostasis in sepsis. Redox Biol. 2025;87:103877. 10.1016/j.redox.2025.103877 PMC12495058 40992080

[21] Jiang H, Liu L, He S, Qu S, Yang Y, Kang G, et al. Dimethyl fumarate reprograms cervical cancer cells to enhance antitumor immunity by activating mtDNA-cGAS-STING pathway. J Biomed Sci. 2025;32:92. 10.1186/s12929-025-01187-x PMC12538808 41116174

[22] Hu W, Hong X, Zhang X, Chen H, Wen X, Lin F, et al. X-ray-responsive dissolving microneedles mediate STING pathway activation to potentiate cutaneous melanoma radio-immunotherapy. Theranostics. 2025;15:6919–37. 10.7150/thno.110841 PMC12203811 40585994

[23] Cao W, Chen G, Wu L, Yu KN, Sun M, Yang M, et al. Ionizing radiation triggers the antitumor immunity by inducing gasdermin E-mediated pyroptosis in tumor cells. Int J Radiat Oncol Biol Phys. 2023;115:440–52. 10.1016/j.ijrobp.2022.07.1841 35918054

[24] Chen R, Zou J, Liu J, Kang R, Tang D. DAMPs in the immunogenicity of cell death. Mol Cell. 2025;85:3874–89. 10.1016/j.molcel.2025.09.007 41106375

[25] Zhou H, Wang X, Xu T, Gan D, Ma Z, Zhang H, et al. PINK1-mediated mitophagy attenuates pathological cardiac hypertrophy by suppressing the mtDNA release-activated cGAS-STING pathway. Cardiovasc Res. 2025;121:128–42. 10.1093/cvr/cvae238 39498806

[26] Jiménez-Loygorri JI, Villarejo-Zori B, Viedma-Poyatos Á, Zapata-Muñoz J, Benítez-Fernández R, Frutos-Lisón MD, et al. Mitophagy curtails cytosolic mtDNA-dependent activation of cGAS/STING inflammation during aging. Nat Commun. 2024;15:830. 10.1038/s41467-024-45044-1 PMC10821893 38280852

[27] Wang R, Shang Y, Chen B, Xu F, Zhang J, Zhang Z, et al. Protein disulfide isomerase blocks the interaction of LC3II-PHB2 and promotes mTOR signaling to regulate autophagy and radio/chemo-sensitivity. Cell Death Dis. 2022;13:851. 10.1038/s41419-022-05302-w PMC9537141 36202782

[28] Pu X, Li X, Liu B, Zhang Y. An AMPK-centric strategy: foenumoside B coordinates PINK1/parkin-mediated mitophagy with Nrf2/HO-1 antioxidant signaling to resolve oxidative stress in radiation-induced lung injury. FASEB J. 2025;39:e71225. 10.1096/fj.202501880R PMC12619048 41239805

[29] Dong Y, Sheng G, Chen W. TPX2 knockdown mediates p53 activation to induce autophagy and apoptosis for anti-colorectal cancer effects. J Recept Signal Transduct Res. 2025;45:129–41. 10.1080/10799893.2025.2470180 40116489

[30] Guirguis DA, Hasan F, Morris N, Alabd A, Tawfik PMS, Pramanik K, et al. Tumor tough, therapy smarter: rethinking CAR-T for pancreatic cancer. Semin Oncol. 2025;52:152411. 10.1016/j.seminoncol.2025.152411 40912036

[31] Lu Z, Zhu W, Liu X. Application prospects of tumor vaccines for pancreatic cancer: from TAAs to TSAs and combination strategies. Semin Oncol. 2025;52:152399. 10.1016/j.seminoncol.2025.152399 40839910

[32] Wu Y, Zhang C, Huang J, Chen Q, Zhang Y, Liu F, et al. Integrated analysis of scRNA-seq and bulk RNA-seq data identifies BHLHE40 as a key gene in pancreatic cancer progression and gemcitabine resistance. Semin Oncol. 2025;52:152338. 10.1016/j.seminoncol.2025.152338 40250076

