# Supplementary material for: Mitophagy-dependent mitochondrial DNA release links ionizing radiation to immunogenic cell death in pancreatic cancer
Source: Cell Death Discov. 2026 Jul 30;12:427. doi: 10.1038/s41420-026-03269-7 (PMC13710541; doi:10.1038/s41420-026-03269-7)

Figure 1

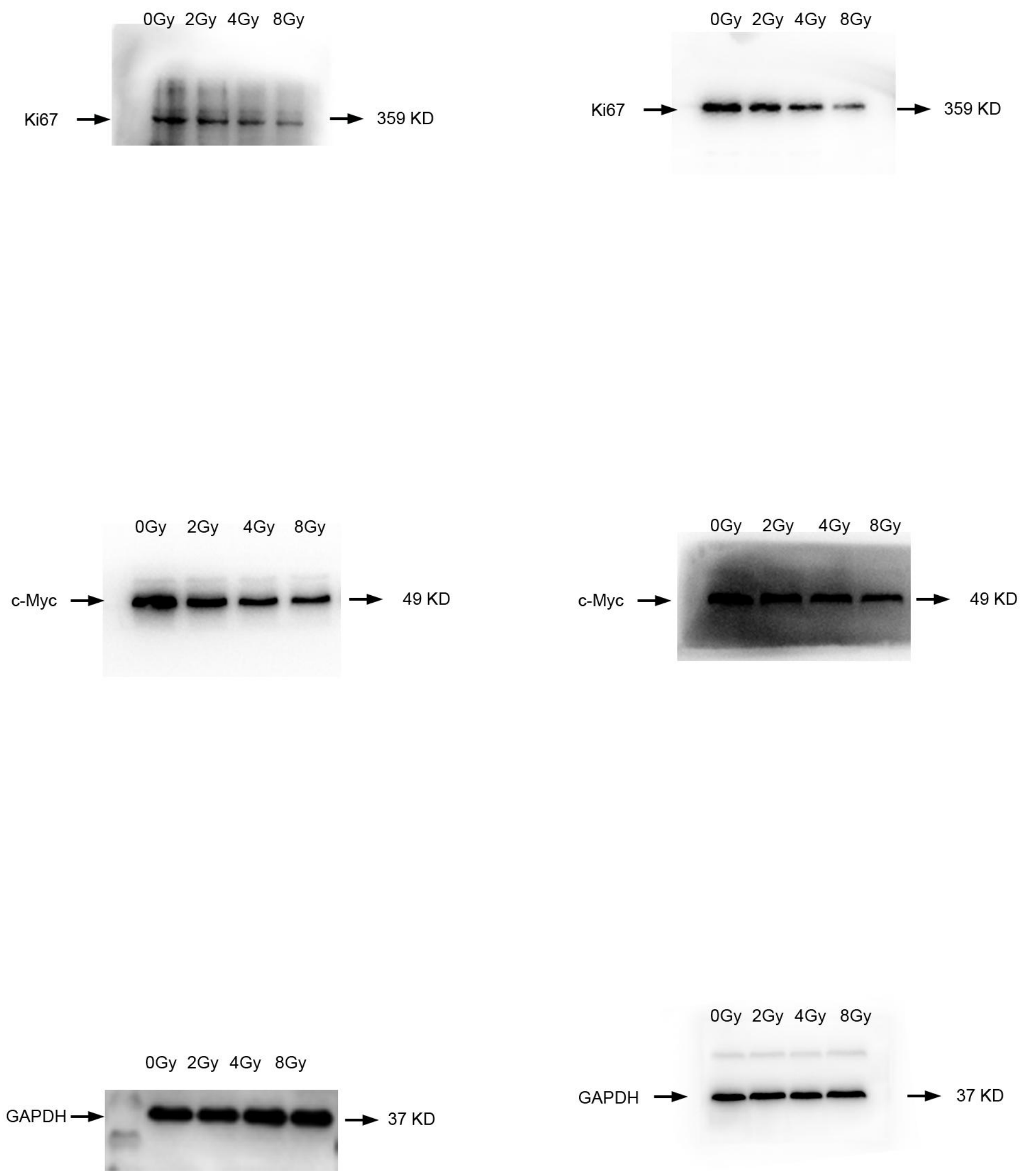
0Gy 2Gy 4Gy 8Gy
Ki67
359 KD
0Gy 2Gy 4Gy 8Gy
Ki67
359 KD
0Gy 2Gy 4Gy 8Gy
c-Myc
49 KD
0Gy 2Gy 4Gy 8Gy
c-Myc
49 KD
0Gy 2Gy 4Gy 8Gy
GAPDH
37 KD
0Gy 2Gy 4Gy 8Gy
GAPDH
37 KD

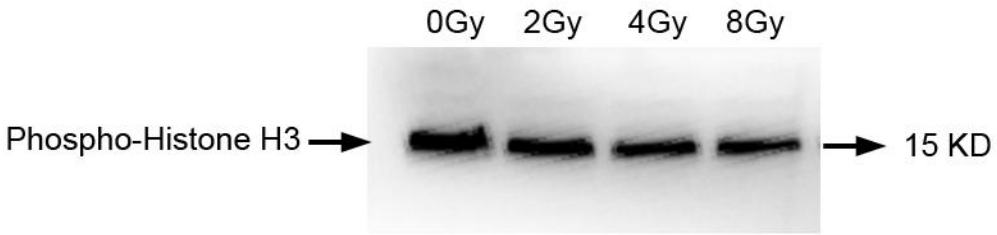
0Gy
2Gy
4Gy
8Gy
Phospho-Histone H3
15 KD

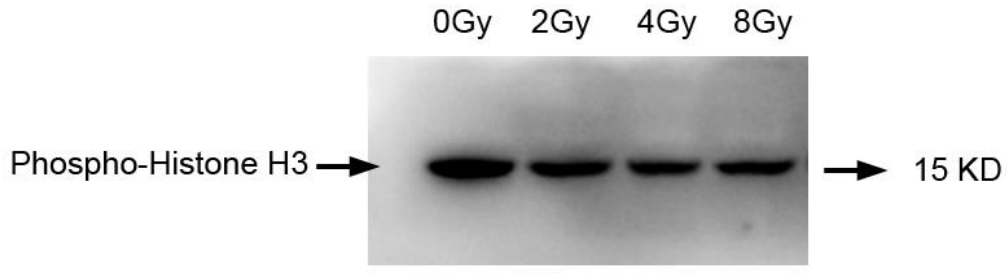
0Gy
2Gy
4Gy
8Gy
Phospho-Histone H3
15 KD

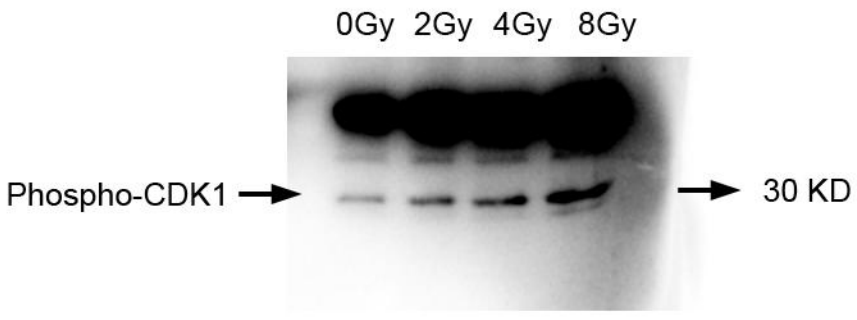
0Gy
2Gy
4Gy
8Gy
Phospho-CDK1
30 KD

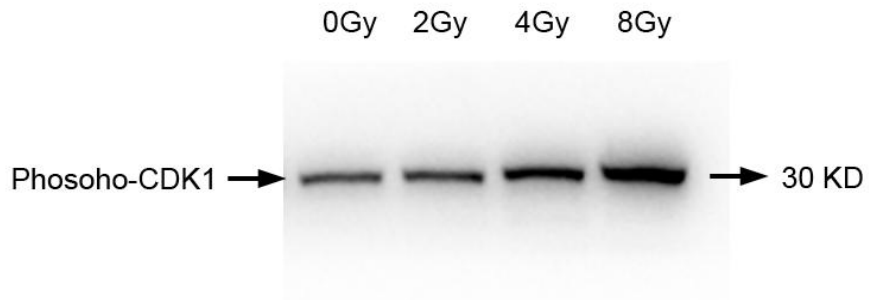
0Gy
2Gy
4Gy
8Gy
Phosoho-CDK1
30 KD

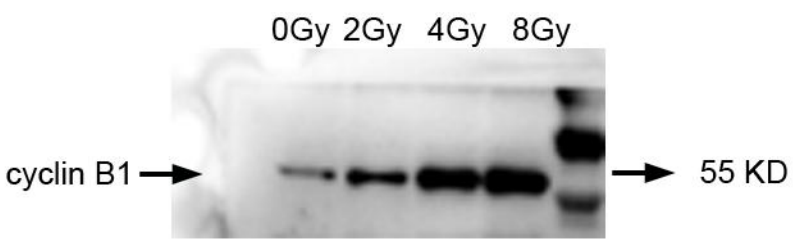
0Gy
2Gy
4Gy
8Gy
cyclin B1
55 KD

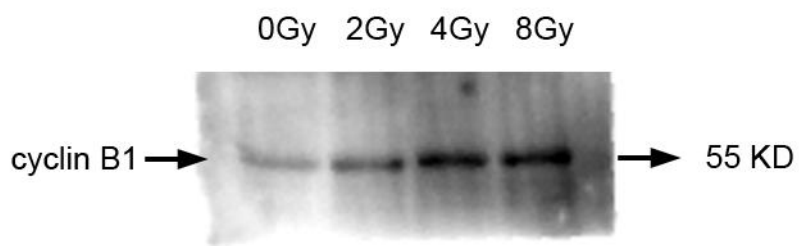
0Gy
2Gy
4Gy
8Gy
cyclin B1
55 KD

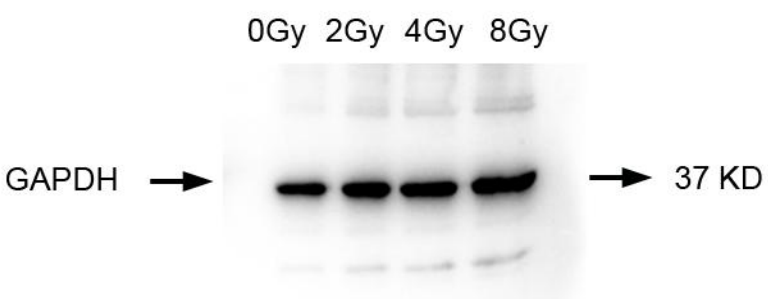
0Gy
2Gy
4Gy
8Gy
GAPDH
37 KD

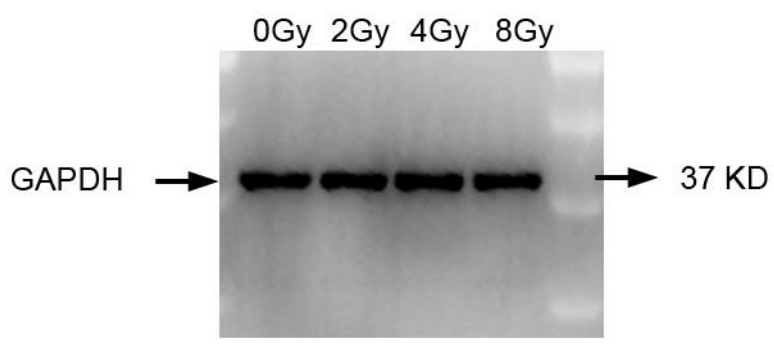
0Gy
2Gy
4Gy
8Gy
GAPDH
37 KD

Figure 2

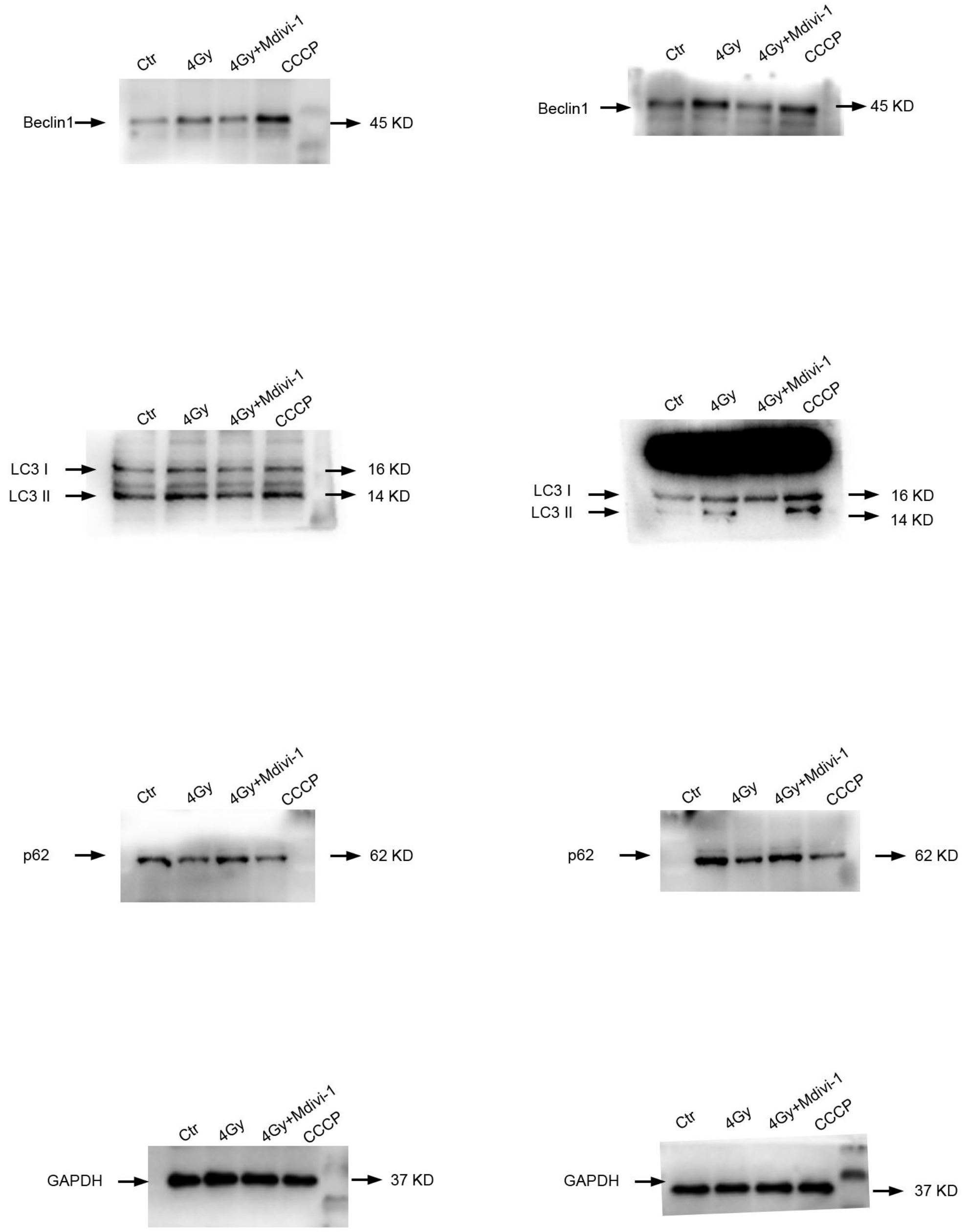

Ctr
4Gy
4Gy+Mdivi-1
CCCP
Beclin1
45 KD
LC3 I
LC3 II
16 KD
14 KD
p62
62 KD
GAPDH
37 KD

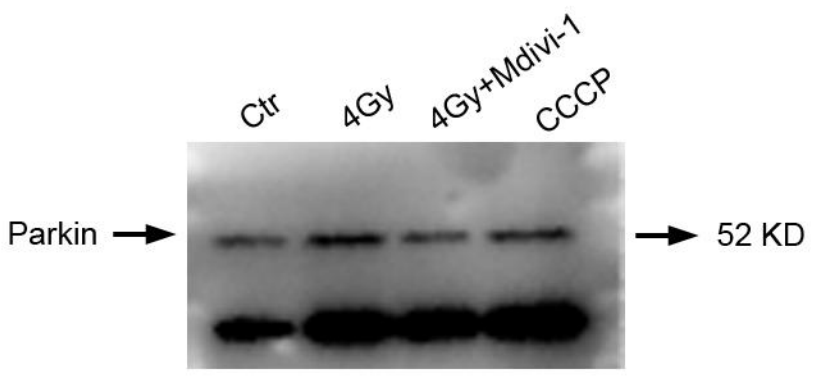
Ctr
4Gy
4Gy+Mdivi-1
CCCP
Parkin
52 KD

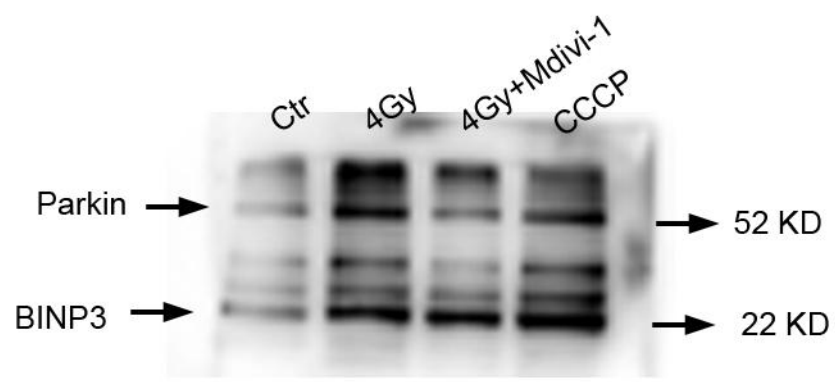
Ctr
4Gy
4Gy+Mdivi-1
CCCP
Parkin
BINP3
52 KD
22 KD

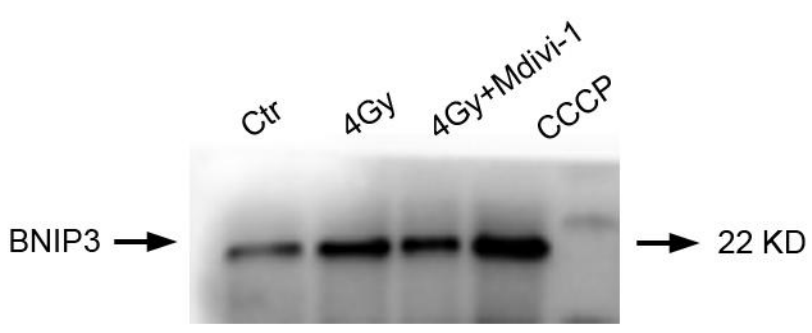
Ctr
4Gy
4Gy+Mdivi-1
CCCP
BNIP3
22 KD

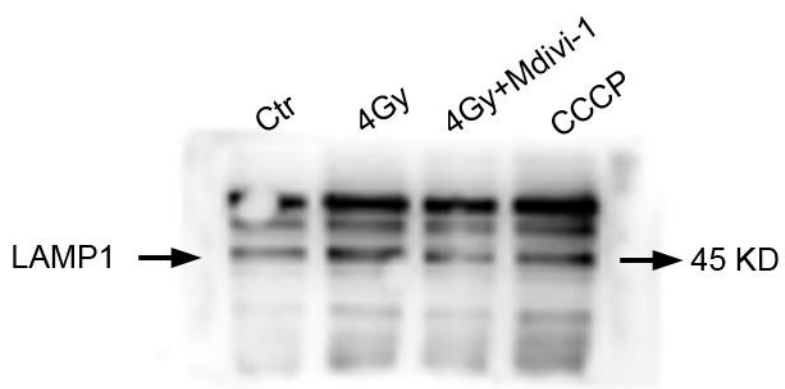
Ctr
4Gy
4Gy+Mdivi-1
CCCP
LAMP1
45 KD

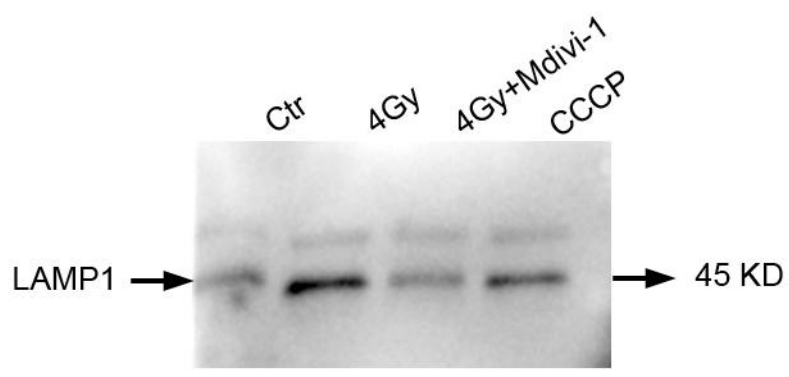
Ctr
4Gy
4Gy+Mdivi-1
CCCP
LAMP1
45 KD

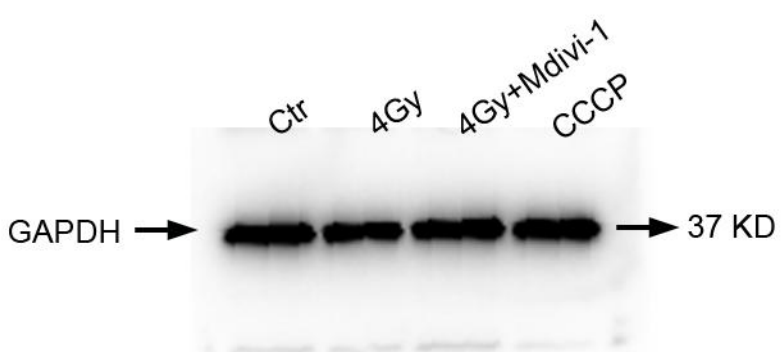
Ctr
4Gy
4Gy+Mdivi-1
CCCP
GAPDH
37 KD

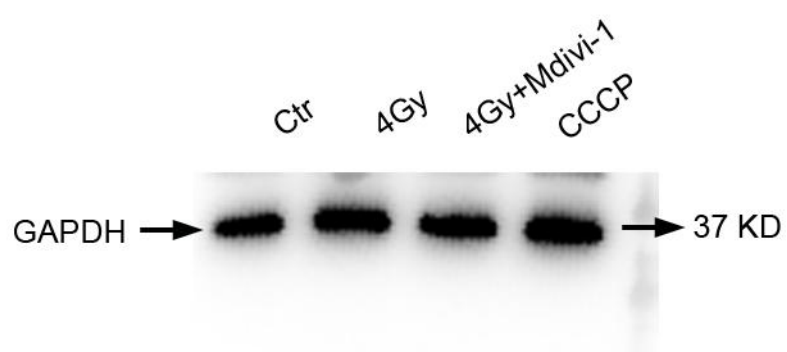
Ctr
4Gy
4Gy+Mdivi-1
CCCP
GAPDH
37 KD

Figure 3

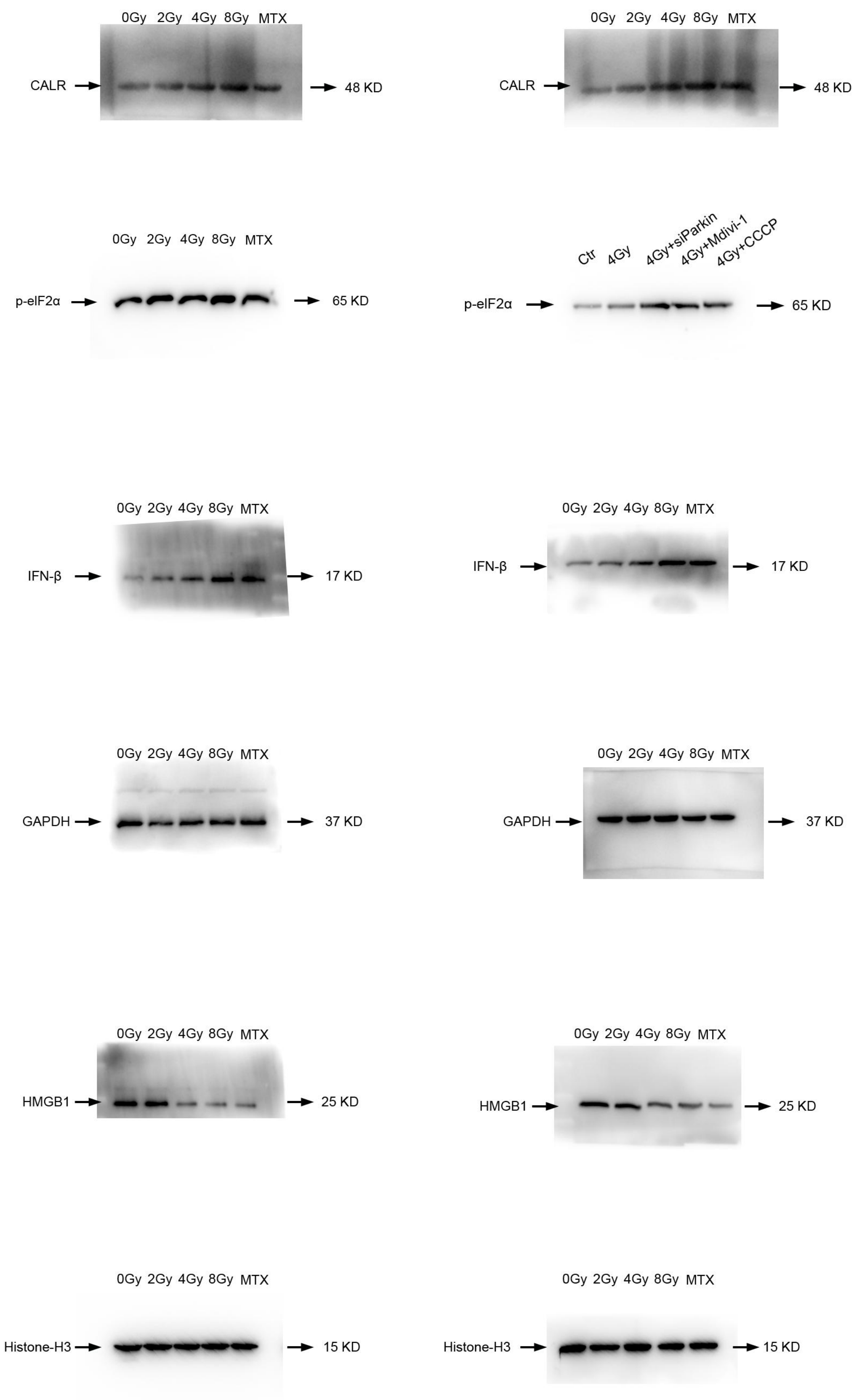
0Gy 2Gy 4Gy 8Gy MTX
CALR
48 KD
0Gy 2Gy 4Gy 8Gy MTX
CALR
48 KD
0Gy 2Gy 4Gy 8Gy MTX
p-eIF2α
65 KD
Ctr 4Gy 4Gy+siParkin 4Gy+Mdivi-1 4Gy+CCCP
p-eIF2α
65 KD
0Gy 2Gy 4Gy 8Gy MTX
IFN-β
17 KD
0Gy 2Gy 4Gy 8Gy MTX
IFN-β
17 KD
0Gy 2Gy 4Gy 8Gy MTX
GAPDH
37 KD
0Gy 2Gy 4Gy 8Gy MTX
GAPDH
37 KD
0Gy 2Gy 4Gy 8Gy MTX
HMGB1
25 KD
0Gy 2Gy 4Gy 8Gy MTX
HMGB1
25 KD
0Gy 2Gy 4Gy 8Gy MTX
Histone-H3
15 KD
0Gy 2Gy 4Gy 8Gy MTX
Histone-H3
15 KD

Figure 4

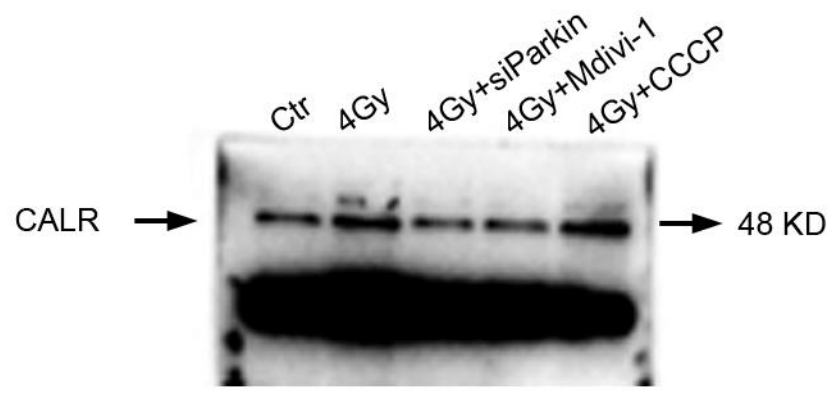
Ctr
4Gy
4Gy+siParkin
4Gy+Mdivi-1
4Gy+CCCP
CALR
48 KD

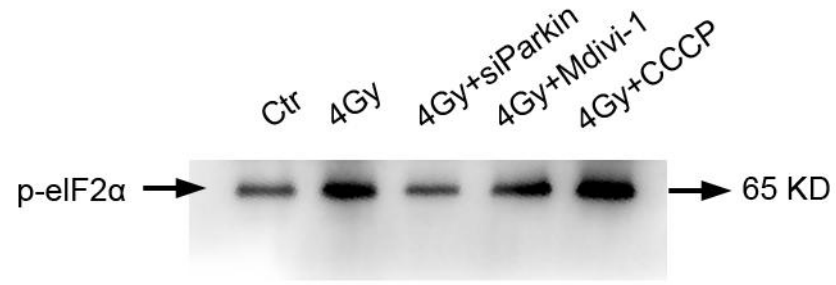
Ctr
4Gy
4Gy+siParkin
4Gy+Mdivi-1
4Gy+CCCP
p-eIF2α
65 KD

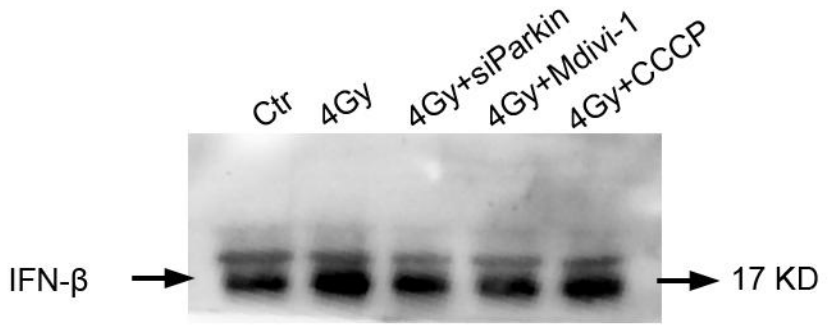
Ctr
4Gy
4Gy+siParkin
4Gy+Mdivi-1
4Gy+CCCP
IFN-β
17 KD

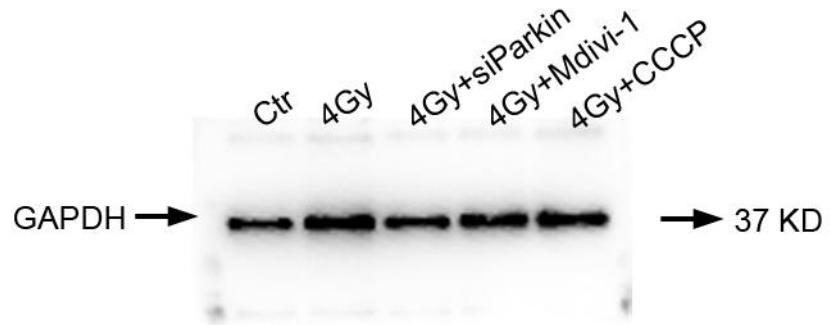
Ctr
4Gy
4Gy+siParkin
4Gy+Mdivi-1
4Gy+CCCP
GAPDH
37 KD

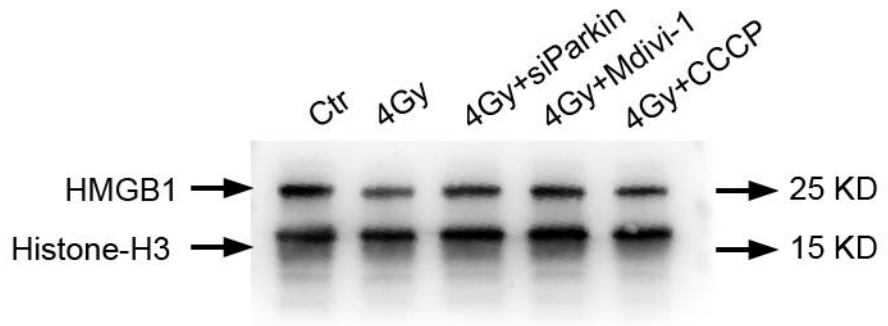
Ctr
4Gy
4Gy+siParkin
4Gy+Mdivi-1
4Gy+CCCP
HMGB1
Histone-H3
25 KD
15 KD

Figure 5

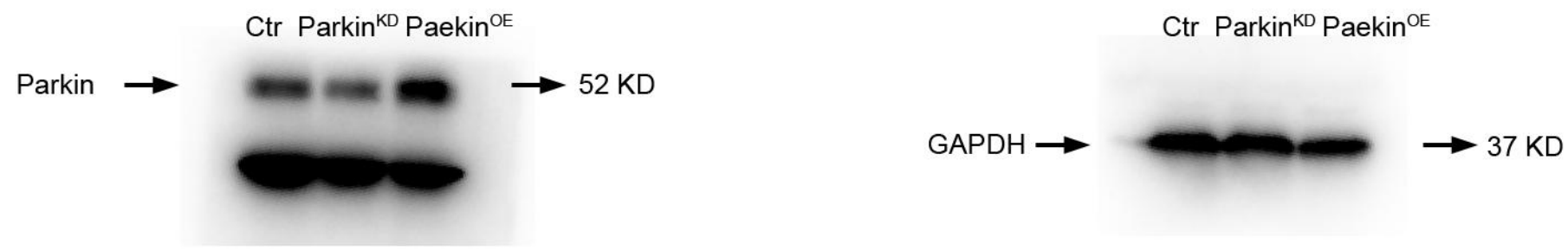
Ctr Parkin^{KD} Paekin^{OE}
Parkin
52 KD
Ctr Parkin^{KD} Paekin^{OE}
GAPDH
37 KD

Figure 6

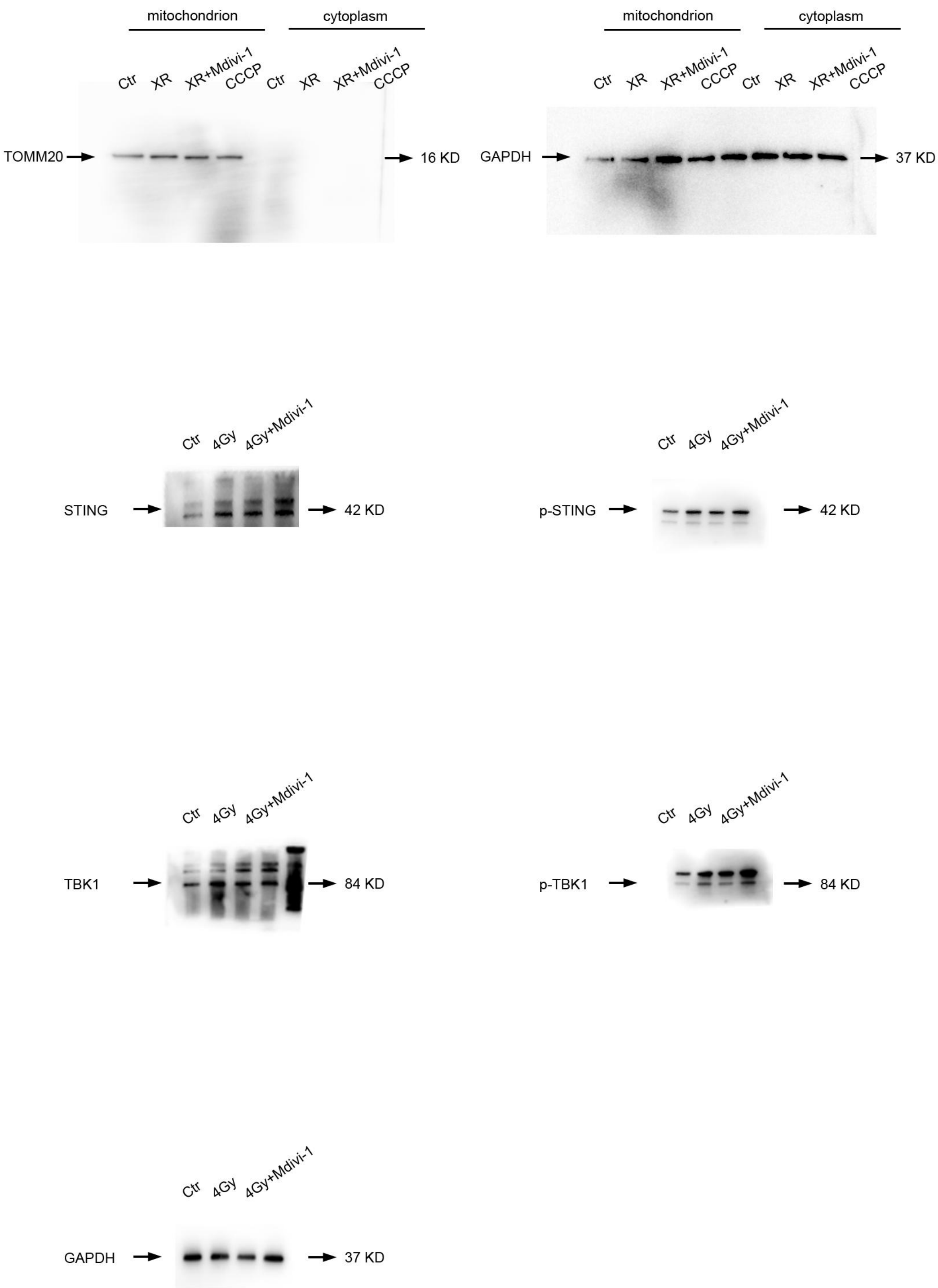
mitochondrion
cytoplasm
Ctr
XR
XR+Mdivi-1
CCCP
Ctr
XR
XR+Mdivi-1
CCCP
TOMM20
16 KD
mitochondrion
cytoplasm
Ctr
XR
XR+Mdivi-1
CCCP
Ctr
XR
XR+Mdivi-1
CCCP
GAPDH
37 KD
Ctr
4Gy
4Gy+Mdivi-1
STING
42 KD
Ctr
4Gy
4Gy+Mdivi-1
p-STING
42 KD
Ctr
4Gy
4Gy+Mdivi-1
TBK1
84 KD
Ctr
4Gy
4Gy+Mdivi-1
p-TBK1
84 KD
Ctr
4Gy
4Gy+Mdivi-1
GAPDH
37 KD

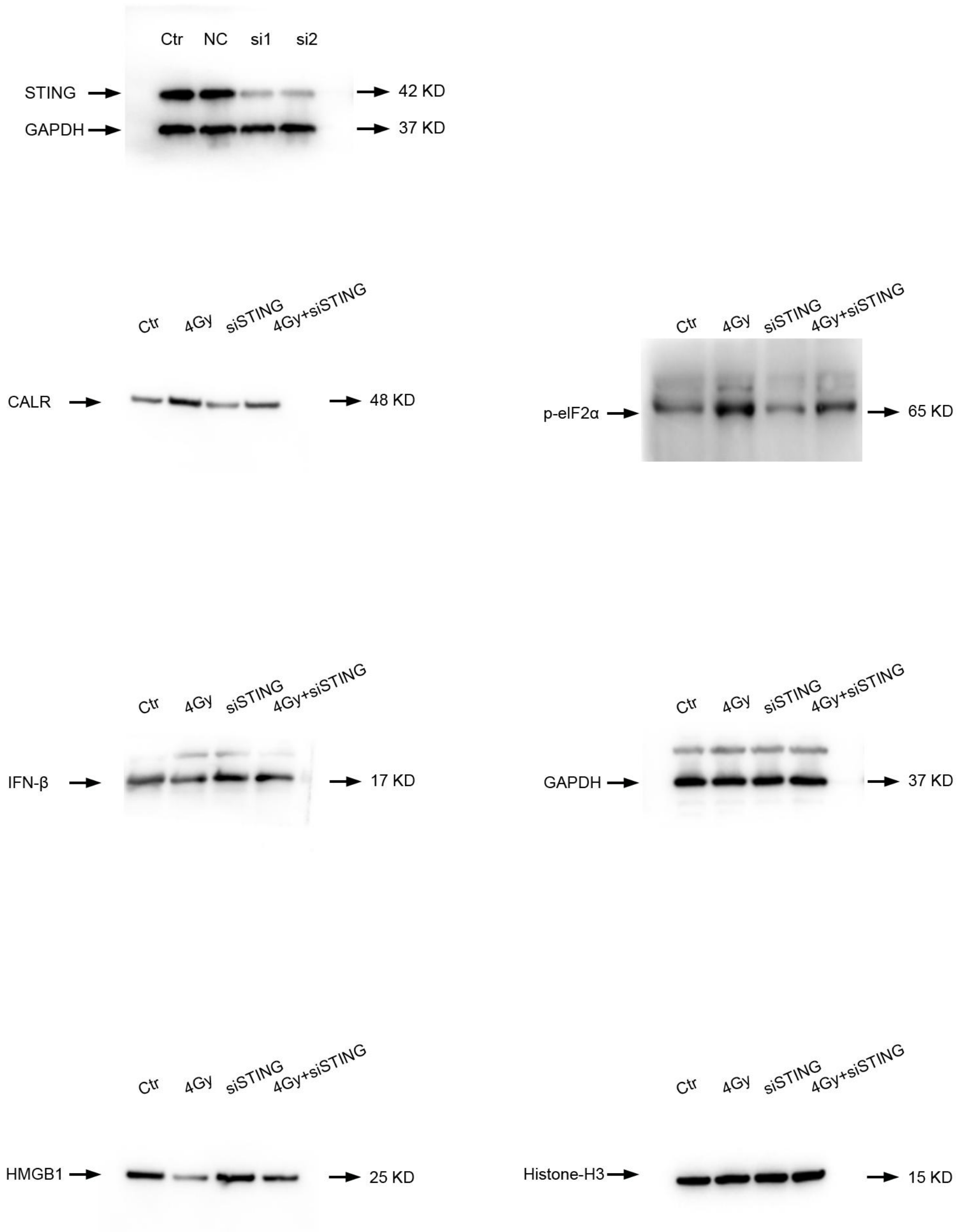
Ctr
NC
si1
si2
STING
42 KD
GAPDH
37 KD
Ctr
4Gy
siSTING
4Gy+siSTING
CALR
48 KD
p-eIF2α
65 KD
IFN-β
17 KD
GAPDH
37 KD
HMGB1
25 KD
Histone-H3
15 KD

Figure 7

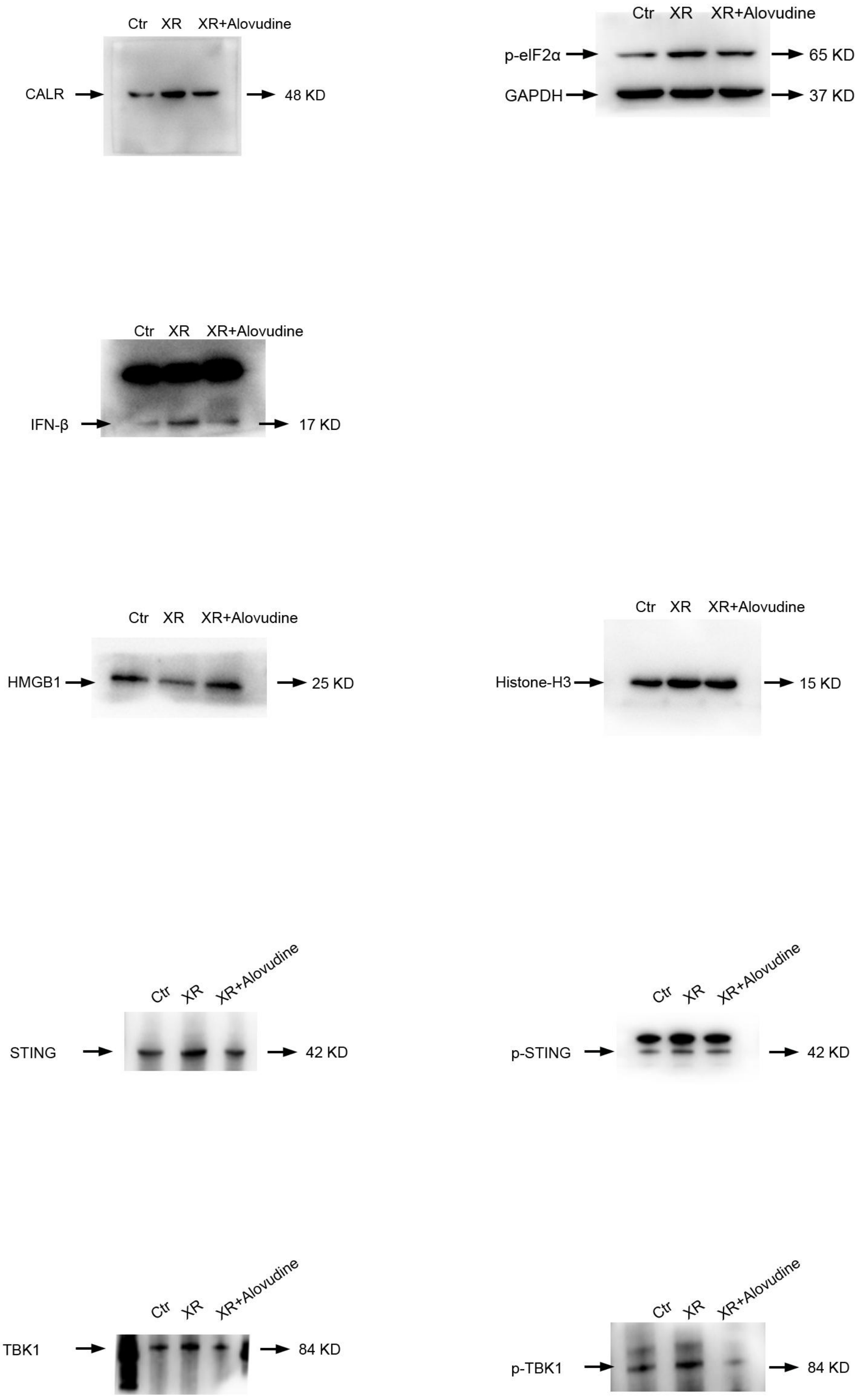
Ctr
XR
XR+Alovudine
CALR
48 KD
p-eIF2α
65 KD
GAPDH
37 KD
IFN-β
17 KD
HMGB1
25 KD
Histone-H3
15 KD
STING
42 KD
p-STING
42 KD
TBK1
84 KD
p-TBK1
84 KD

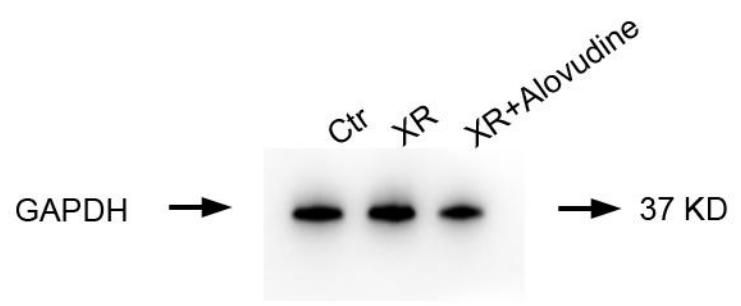
Ctr
XR
XR+Alovudine
GAPDH
37 KD

Figure S1

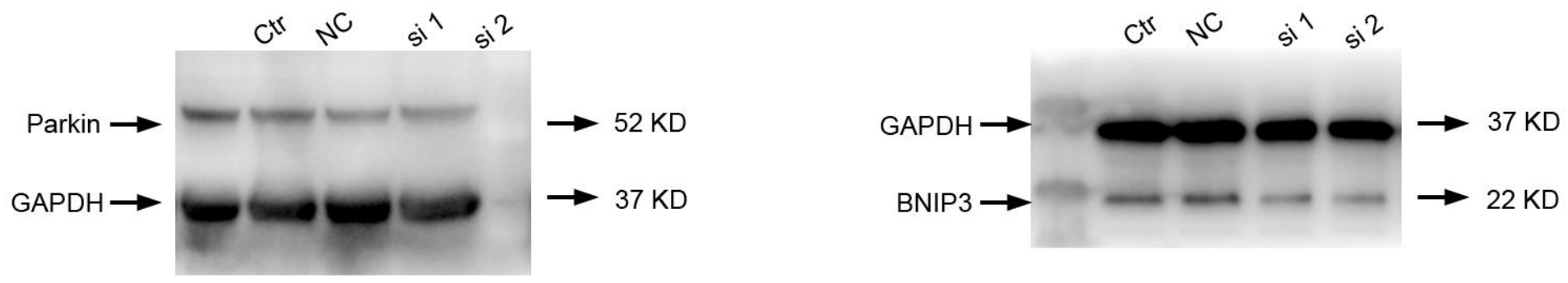

Figure S2

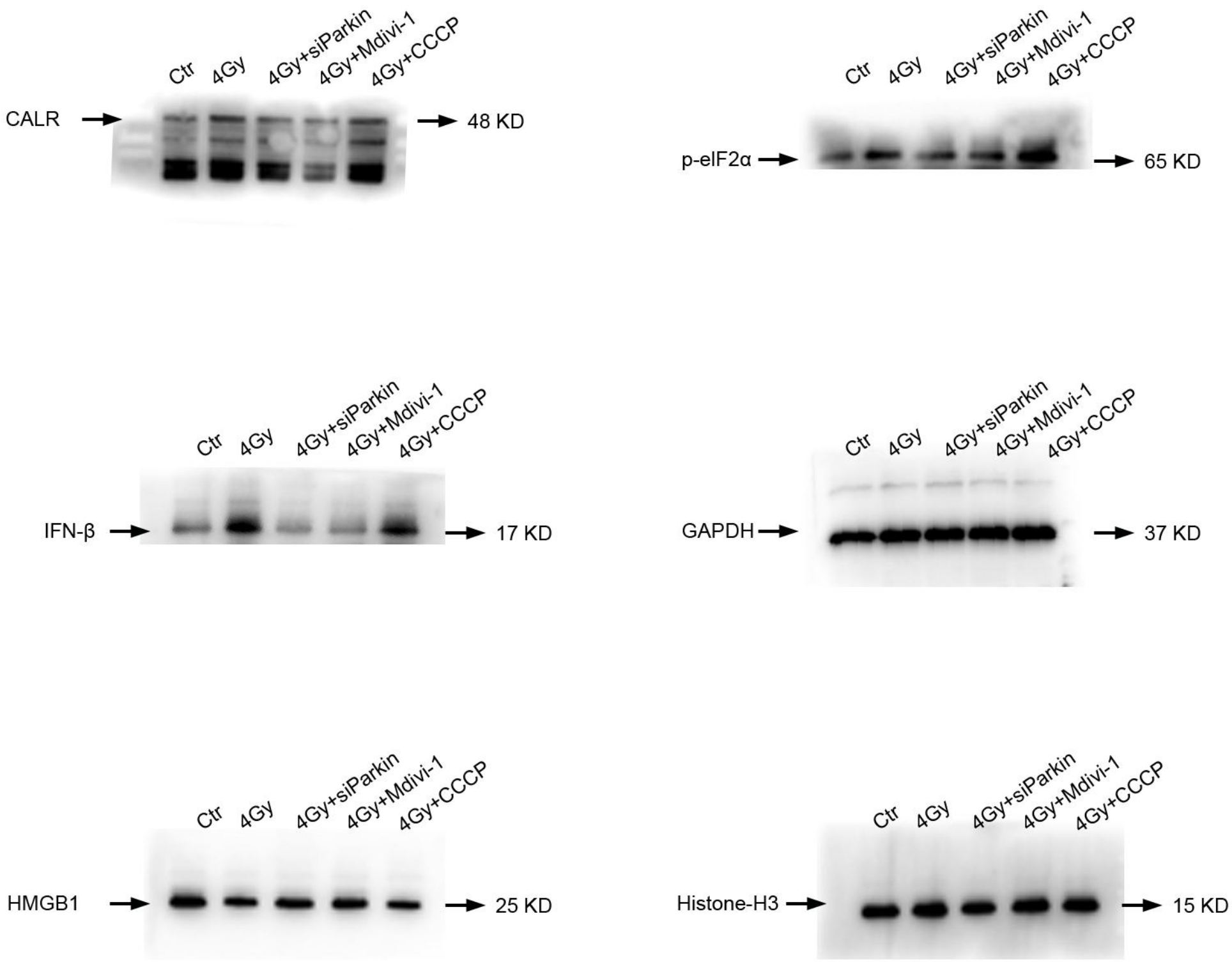

Supplement: Supplementary file 1 [file 41420_2026_3269_MOESM8_ESM.pdf]
