# Supplementary material for: Mitophagy-dependent mitochondrial DNA release links ionizing radiation to immunogenic cell death in pancreatic cancer
Source: Cell Death Discov. 2026 Jul 30;12:427. doi: 10.1038/s41420-026-03269-7 (PMC13710541; doi:10.1038/s41420-026-03269-7)
Supplement: Supplementary file 2 [file 41420_2026_3269_MOESM5_ESM.docx]

**Supplementary Figure Legends**

**Figure S1. Immunofluorescence analysis of LC3B expression in pancreatic cancer cells.** (A, B) Representative immunofluorescence images showing LC3B staining in PANC-1 and SW1990 cells after 4 Gy irradiation or CCCP treatment, while Mdivi-1 co-treatment attenuates these changes. Nuclei were counterstained with DAPI. Data are expressed as mean ± SD. *P < 0.05, **P < 0.01, ***P < 0.001. (C, D) Western blot confirming reduced protein expression of Parkin and BNIP3 following siRNA transfection in PANC-1 cells.

**Figure S2. Mitophagy regulates radiation-induced ICD marker expression.** (A–C) Immunofluorescence staining showing that radiation-induced CALR exposure, eIF2α phosphorylation, IFN-β expression and HMGB1 nuclear export are diminished by mitophagy inhibition (siRNA BNIP3 or Mdivi-1) and enhanced by VPA. (D) ELISA detection of ATP and IFN-β release confirming that mitophagy inhibition suppresses, while VPA enhances, radiation-induced secretion. (E) Western blot validation of ICD marker modulation by mitophagy inhibition or induction, consistent with immunofluorescence and ELISA results. Data are expressed as mean ± SD. *P < 0.05, **P < 0.01, ***P < 0.001.

**Figure S3. Prognostic value and immune infiltration of PRKN in pancreatic cancer based on the TCGA database.** (A) Correlation between PRKN expression and pancreatic cancer occurrence. (B, C) Kaplan-Meier survival curves showing disease-specific survival (DSS) and overall survival (OS) in patients with high versus low PRKN expression. (D, E) Infiltration levels of various immune cell populations in pancreatic cancer tissues with high versus low Parkin expression. (F, G) Distribution of Stromal Score, Immune Score, and ESTIMATE Score across different Parkin expression groups.

**Figure S4. Parkin overexpression potentiates local and systemic antitumor immunity.** (A–D) Representative immunohistochemistry images and quantification of CD8, IFN-γ, Granzyme B, and Perforin expression in excised tumor tissues. (E–H) Flow cytometric analysis of CD8^+^ T cells and the expression levels of IFN-γ, Granzyme B, and Perforin in peripheral blood from the indicated groups. Data are expressed as mean ± SD. *P < 0.05, **P < 0.01, ***P < 0.001.
