# Supplementary material for: Mitophagy-dependent mitochondrial DNA release links ionizing radiation to immunogenic cell death in pancreatic cancer
Source: Cell Death Discov. 2026 Jul 30;12:427. doi: 10.1038/s41420-026-03269-7 (PMC13710541; doi:10.1038/s41420-026-03269-7)
Supplement: Supplementary file 3 [file 41420_2026_3269_MOESM6_ESM.docx]

**Table S1.** **List of primers and sequence**

| Name Sequence | |
| --- | --- |
| Parkin siRNA(h) (sc-42158)  Parkin shRNA(m) (sc-42159)  BNIP3 siRNA(h) (sc-37451)  BNIP3 shRNA(m) (sc-37452)  STING sgRNA1 (P62486)  STING sgRNA2 (P62487) | F：5′-CUA CCC AGU GAC CAU GAU Att-3′  R：5′-UAU CAU GGU CAC UGG GUA Gtt-3′  F：5′-CUA CAG AGU CGA UGA AAG Att-3′  R：5′-UCU UUC AUC GAC UCU GUA Gtt-3′  F：5′-GGA UUG GAU UUC AGU UCA Att-3′  R：5′-UUG AAC UGA AAU CCA AUC Ctt-3′  F：5′-GGA ACA ACA GAG UAU UGU Att-3′  R：5′-UAC AAU ACU CUG UUG UUC Ctt-3′  F：5′- CGA UGC AUA UAA GCA CAU Att-3′  R：5′- UAU GUG CUU AUA UGC AUC Gtt-3′  F：5′-CUU GAG GCC UAG AGC UAA Att-3′  R：5′-UUU AGC UCU AGG CCU CAA Gtt-3′  F：5′-GAA CUG CAC UUC AGC AAU Att-3′  R：5′-UAU UGC UGA AGU GCA GUU Ctt-3′  F：5′-CCA UAG CAU UGG AGA GAA Att-3′  R：5′-UUU CUC UCC AAU GCU AUG Gtt-3′  F：5′-GAA GGC ACC UAC UCA GUA Utt-3′  R：5′-AUA CUG AGU AGG UGC CUU Ctt-3′  F：5′-CCA ACA GAG CUG AAA UAG Att-3′  R：5′-UCU AUU UCA GCU CUG UUG Gtt-3′  F：5′-CUC UUG UCA UGA CCU AUA Att-3′  R：5′-UUA UAG GUC AUG ACA AGA Gtt-3′  F：5′- CCA GUU AAG ACA CUU UAC Att-3′  R：5′-UGU AAA GUG UCU UAA CUG Gtt-3′  5′-GCTGGGACTGCTGTTAAACG-3′  5′-CATATTACATCGGATATCTG-3′ |
