# Supplementary material for: Mitophagy-dependent mitochondrial DNA release links ionizing radiation to immunogenic cell death in pancreatic cancer
Source: Cell Death Discov. 2026 Jul 30;12:427. doi: 10.1038/s41420-026-03269-7 (PMC13710541; doi:10.1038/s41420-026-03269-7)
Supplement: Supplementary file 4 [file 41420_2026_3269_MOESM7_ESM.docx]

**Table S2.** List of primers and sequence

| Name Sequence | |
| --- | --- |
| B2M  ND1 | F：5′-TGCTGTCTCCATGTTTGATGTATCT-3′  R：5′-TCTCTGCTCCCCACCTCTAAGT-3′  F：5′-CCCTAAAACCCGCCACATCT-3′  R：5′-GAGCGATGGTGAGAGCTAAGGT-3′ |
